# RAC1^P29S^ Induces a Mesenchymal Phenotypic Switch via Serum Response Factor to Promote Melanoma Development and Therapy Resistance

**DOI:** 10.1016/j.ccell.2019.05.015

**Published:** 2019-07-08

**Authors:** Daniël A. Lionarons, David C. Hancock, Sareena Rana, Philip East, Christopher Moore, Miguel M. Murillo, Joana Carvalho, Bradley Spencer-Dene, Eleanor Herbert, Gordon Stamp, Djamil Damry, Dinis P. Calado, Ian Rosewell, Ralph Fritsch, Richard R. Neubig, Miriam Molina-Arcas, Julian Downward

**Affiliations:** 1 Oncogene Biology, Francis Crick Institute, 1 Midland Road, London NW1 1AT, UK; 2 Bioinformatics & Biostatistics, Francis Crick Institute, 1 Midland Road, London NW1 1AT, UK; 3 Experimental Histopathology, Francis Crick Institute, 1 Midland Road, London NW1 1AT, UK; 4 Immunity & Cancer Laboratories, Francis Crick Institute, 1 Midland Road, London NW1 1AT, UK; 5 Genetic Manipulation Service, Francis Crick Institute, 1 Midland Road, London NW1 1AT, UK; 6 Lung Cancer Group, Division of Molecular Pathology, Institute of Cancer Research, 237 Fulham Road, London SW3 6JB, UK; 7 Royal Veterinary College, Hatfield AL9 7TA, UK; 8 Universitätsklinikum Freiburg, Freiburg 79106, Germany; 9 Michigan State University, East Lansing, MI 48824, USA

**Keywords:** RAC1, melanoma, SRF, MRTF, EMT, BRAF, NF1, PTEN, p53

## Abstract

*RAC1* P29 is the third most commonly mutated codon in human cutaneous melanoma, after *BRAF* V600 and *NRAS* Q61. Here, we study the role of RAC1^P29S^ in melanoma development and reveal that RAC1^P29S^ activates PAK, AKT, and a gene expression program initiated by the SRF/MRTF transcriptional pathway, which results in a melanocytic to mesenchymal phenotypic switch. Mice with ubiquitous expression of RAC1^P29S^ from the endogenous locus develop lymphoma. When expressed only in melanocytes, RAC1^P29S^ cooperates with oncogenic BRAF or with *NF1*-loss to promote tumorigenesis. RAC1^P29S^ also drives resistance to BRAF inhibitors, which is reversed by SRF/MRTF inhibitors. These findings establish RAC1^P29S^ as a promoter of melanoma initiation and mediator of therapy resistance, while identifying SRF/MRTF as a potential therapeutic target.

## Highlights

• RAC1^P29S^ activates PAK, AKT, and the SRF/MRTF transcription program in melanocytes
• RAC1^P29S^ induces a melanocytic to mesenchymal transition through SRF/MRTF and PAK
• RAC1^P29S^ cooperates with BRAF mutation or NF1 deletion to promote melanomagenesis
• RAC1^P29S^ induces resistance to BRAF inhibitors through SRF/MRTF

## Graphical Abstract

**Figure:**
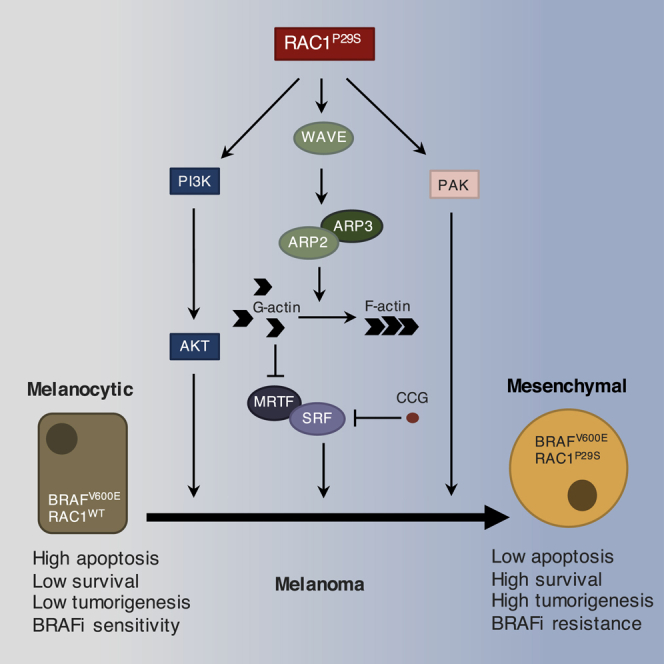

## Teaser

*RAC1*^P29S^ is a common mutation in human cutaneous melanoma. Lionarons et al. show that RAC1^P29S^ induces a melanocytic to mesenchymal switch via an SRF/MRTF-mediated gene expression program, cooperates with BRAF in melanomagenesis, and drives BRAF inhibitor resistance, which is reversed by SRF/MRTF inhibition.

## Significance

**Metastatic melanoma is a lethal disease, partly due to rapid acquisition of resistance to therapy. Using genetically engineered mouse models, we demonstrate that the activating RAC1**^**P29S**^ **mutation, present in up to 4% of melanoma cases, cooperates with BRAF as a driver of melanoma initiation and promotes BRAF inhibitor resistance. Since clinical inhibitors of RAC1 are not currently available, we systematically evaluated RAC1**^**P29S**^ **signaling and the functional significance of its downstream effector pathways. Our findings suggest that the critical RAC1 effector in melanoma is the transcription factor complex SRF/MRTF, which initiates a switch to a mesenchymal-like state characterized by therapy resistance. Therapeutic targeting of SRF/MRTF may have potential to reverse BRAF inhibitor resistance in melanoma patients bearing the oncogenic RAC1**^**P29S**^ **mutation.**

## Introduction

Melanoma is a potentially lethal form of skin cancer responsible for the death of 55,500 people per year globally, with incidence rising rapidly, especially in Western countries (Eggermont et al., 2014). Chronic sun-exposure is the most important cause of melanoma, with UV radiation-induced mutations, particularly in tumor suppressor genes, contributing to the development of the disease (Viros et al., 2014). The commonest oncogenic driver mutation is *BRAF*^V600E^, present in up to 50% of cases, followed by *NRAS* codon 61, mutated in about 20% of melanomas. The third most common mutation lies in the gene encoding the RHO family small GTPase RAC1. The *RAC1* P29S mutation is present in around 4% of melanomas. This amino acid change arises from a UV radiation-induced C > T transition at a dipyrimidine site. The P29S mutation activates the biological activity of RAC1 by promoting guanine nucleotide exchange, hence increasing the relative ratio of active GTP-bound RAC1 to inactive GDP-bound RAC1 (Hodis et al., 2012, Krauthammer et al., 2012). The potential importance of RAC1 function in melanoma is further underlined by the discovery of frequent alterations in PREX2, and to a lesser extent PREX1, which are guanine nucleotide exchange factors for RAC1 (Berger et al., 2013). In at least some cases, truncating mutations in PREX2 have been shown to result in gain of function in terms of RAC1 activation (Lissanu Deribe et al., 2016). Overall, 30% of melanomas show alterations in either PREX1 or PREX2, although the functional significance of most of these individual changes has not been tested and may not all be mediated through RAC1 signaling.

Despite frequent mutational activation of RAC1 and its regulators in melanoma, the functional implications for the disease are poorly understood. As expected for a fast-cycling mutant protein, RAC1^P29S^ has been reported to show increased binding to direct effectors such as PAK1, MLK3, and the WAVE complex (Chen et al., 2017, Hodis et al., 2012, Kawazu et al., 2013, Krauthammer et al., 2012, Watson et al., 2014). Overexpression of RAC1^P29S^ increased cell proliferation, migration, anchorage-independent growth, and promoted growth of transplanted cells in nude mice. Varying and relatively modest effects of RAC1^P29S^ on MAPK activation have been reported. In addition, RAC1^P29S^ has been reported to promote the expression of the immune checkpoint ligand PD-L1 in cells (Vu et al., 2015). Finally, it was reported that ectopic expression of RAC1^P29S^ in BRAF mutant melanoma cells generates resistance to BRAF inhibition and that three patients with the mutation responded particularly poorly to a BRAF inhibitor (Van Allen et al., 2014).

Genetically engineered mouse models addressing the role of RAC1 in melanomagenesis have yet to be reported. However, deletion of *Prex1*, encoding an upstream activator of RAC1, has been reported to suppress migration and metastasis in a melanoma model (Lindsay et al., 2011). In addition, mice with inducible overexpression of the activated PREX2^E824∗^ mutant in melanocytes showed increased melanomagenesis (Lissanu Deribe et al., 2016).

Overall, there is a considerable body of evidence pointing to an important role for RAC1 signaling in melanoma, but the nature of its function remains largely unclear. Which downstream effectors of RAC1 might be important in melanoma is not known, although a recent report has highlighted a potential role for PAK in a zebrafish model and in human melanoma cell lines (Araiza-Olivera et al., 2018). A greater understanding of the role of RAC1^P29S^ in melanoma and the effector pathways involved may provide therapeutic targets.

## Results

### Expression of RAC1^P29S^ Promotes Cell Survival in the Absence of Growth Factors or Anchorage

To investigate RAC1^P29S^ signaling, we stably expressed RAC1^WT^, RAC1^P29S^, and the constitutively active mutant RAC1^Q61L^ in MCF10A immortalized breast epithelial cells (Figure S1A). RAC1 codon 61 mutations are not found in human melanoma, but RAC1^Q61L^ served as a control for increased RAC1 signaling. RAC1^P29S^ promoted survival when cells were starved of growth factors, whereas proliferation in normal medium was unaffected (Figures S1B and S1C). When seeded in soft agar, RAC1^P29S^ enabled the formation of spheres (Figure S1D). Constitutively active RAC1^Q61L^ promoted sphere formation to a lesser extent than RAC1^P29S^, while suppressing the 2D proliferation rate, which may explain why this mutation is not found in human melanoma.

**Figure 1:**
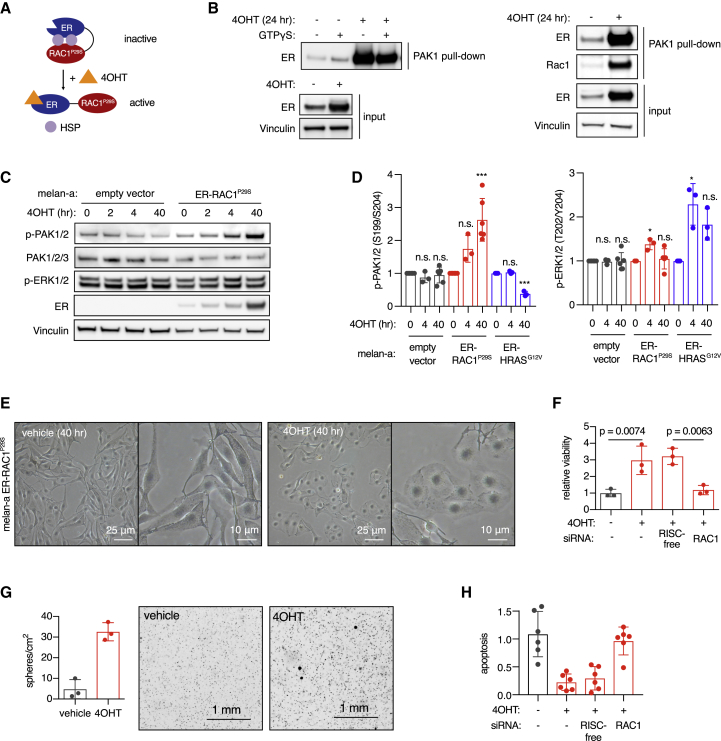
Effect of Activation of RAC1^P29S^ on Survival of Melanocytes (A) Schematic of the ER-RAC1^P29S^ fusion protein system. ER, estrogen receptor; 4OHT, 4-hydroxytamoxifen; HSP, heat shock protein. (B) RAC1-GTP assay in MCF10A cells (left) and mouse melanocyte cell line melan-a (right) stably expressing ER-RAC1^P29S^. Cells were treated with 500 nM 4OHT for 24 h and ER-RAC1^P29S^ binding to the PAK1 RAC1 binding domain was assessed using a pull-down assay. (C) PAK1/2 and ERK1/2 phosphorylation by ER-RAC1^P29S^ in melanocytes. Cells were treated with 500 nM 4OHT for 24 h. (D) Quantification of phospho-PAK1/2 and phospho-ERK1/2 levels detected by immunoblotting, normalized using vinculin loading control (n = 3 or more independent experiments); t test versus 0 h was used with Holm-Sidak correction for multiple testing; ^∗^p < 0.05, ^∗∗∗^p < 0.001; n.s., not statistically significant. (E) Morphological changes induced by ER-RAC1^P29S^ activation in melan-a melanocytes treated with 500 nM 4OHT. (F) Viability of melan-a melanocytes with ER-RAC1^P29S^ in growth factor-reduced medium (fetal calf serum [FCS] 0.25%, 12-O-tetradecanoylphorbol-13-acetate [TPA]-free). Cells were treated with 1 μM 4OHT and viability was quantified using CellTiter-Glo (n = 3 independent experiments); t test was used for statistical comparison. (G) Soft agar sphere formation of melan-a melanocytes with activated ER-RAC1^P29S^. Cells were grown in full medium containing 1 μM 4OHT for 3 weeks before staining, imaging, and automated counting (n = 3 biological replicates). (H) Effect of activation of ER-RAC1^P29S^ on apoptosis in melan-a melanocytes. Cells were treated with 1 μM 4OHT. Cleaved caspase-3 was quantified and normalized to cell viability (n = 2 independent experiments, three biological replicates per experiment). Bars represent means ± SD. See also Figure S1.

To study the signaling effects of RAC1^P29S^ in a temporally controlled manner, we developed an ER-RAC1^P29S^ fusion protein system that can be acutely activated by treatment with 4-hydroxytamoxifen (4OHT) (Figure 1A). Activation of ER-RAC1^P29S^ led to increased binding and phosphorylation of the RAC1 effector PAK1 in both MCF10A cells and the mouse immortalized melanocyte cell line melan-a (Figures 1B, 1C and S1E), but caused extracellular signal-regulated kinase (ERK) activation only transiently and weakly (Figures 1C, 1D, S1E, and S1F), in contrast to ER-RAC1^Q61L^ (Figure S1E). Differential activation of the MAPK pathway was not associated with ER-RAC1 protein expression levels, which were similar (Figure S1G). Melanocytes with ER-RAC1^P29S^ underwent a morphological change upon 4OHT treatment, with generalized extension of lamellipodia (Figure 1E) and increased peripheral staining of polymerized actin (Figure S1H). Activation of ER-RAC1^P29S^ in melanocytes promoted survival when cells were starved of growth factors and enabled sphere formation when seeded in soft agar (Figures 1F and 1G), with only minor effects on proliferation in 2D culture (Figure S1I). In low adhesion conditions, activated ER-RAC1^P29S^ promoted cell survival (Figure S1J). When starved of growth factors, activated ER-RAC1^P29S^ suppressed apoptosis (Figure 1H). Knockdown of RAC1 reversed the effects on viability and apoptosis (Figures 1F–1H).

We have thus developed an ER fusion protein system that allows temporal control of RAC1^P29S^ activation in melanocytes, revealing that RAC1^P29S^ protects against apoptosis and provides cells with a survival advantage when deprived of growth factors or anchorage.

### RAC1^P29S^ Activates an SRF/MRTF Transcriptional Program and Drives Melanocytes toward a Mesenchymal State

**Figure 2:**
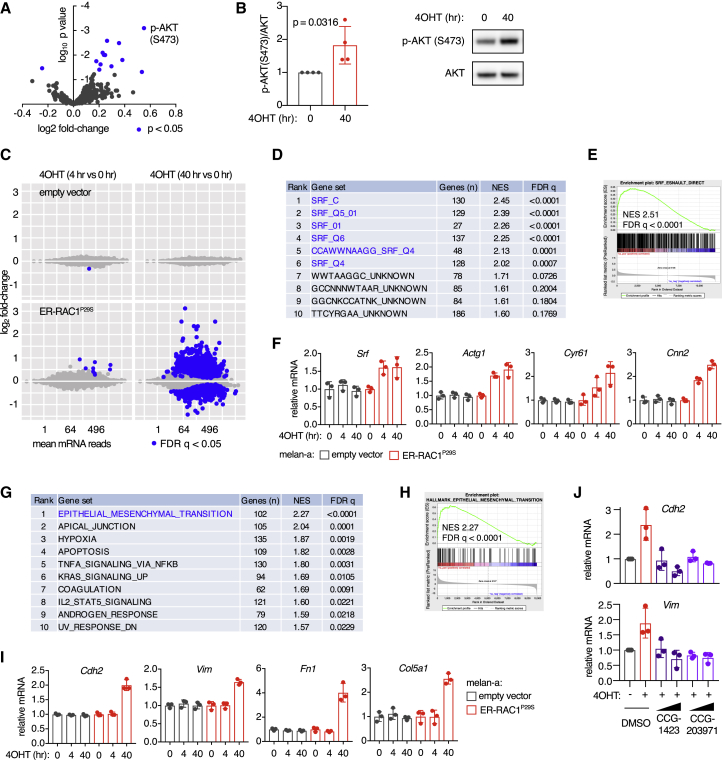
Effect of Acute Activation of RAC1^P29S^ in Melanocytes on AKT and SRF/MRTF Effector Pathways (A) Reverse-phase protein array using ER-RAC1^P29S^ melanocytes after RAC activation, expressed as a scatterplot with p values and log2 fold change compared with 0 h control (means from 3 independent experiments). (B) Densitometric quantification of phospho-AKT levels determined by immunoblotting, normalized to total AKT levels. Melan-a ER-RAC1^P29S^ cells were treated with 4OHT at 500 nM (n = 4 independent experiments, one representative immunoblot shown); t test was used for statistical comparison. (C) RNA sequencing (RNA-seq) in ER-RAC1^P29S^ melanocytes, expressed as scatterplots for empty vector cells and ER-RAC1^P29S^ cells treated with 500 nM 4OHT. Blue dots represent values statistically different to 0 h values (false discovery rate [FDR] 5%). (D) Transcription factor target enrichment analysis of gene expression changes in melanocytes 4 h after activation of ER-RAC1^P29S^. Gene set enrichment analysis (GSEA) using the transcription factor target gene sets from the MSigDB database (Broad). NES, normalized enrichment score. (E) GSEA plot of the direct SRF target gene set from Esnault et al. (2014), using all genes expressed in the ER-RAC1^P29S^ melanocytes at 4 versus 0 h 4OHT treatment. (F) Normalized RNA-seq read counts of canonical SRF/MRTF targets (n = 3 independent experiments). (G) Pathway enrichment analysis of gene expression changes in melanocytes 40 h after activation of ER-RAC1^P29S^. GSEA using the curated hallmark gene set collection from MSigDB. (H) GSEA plot of the epithelial-to-mesenchymal transition (EMT) hallmark gene set from MSigDB using gene expression differences in ER-RAC1^P29S^ melanocytes treated for 40 h with 4OHT compared with 0 h control. (I) Relative mRNA of mesenchymal markers in melanocytes with activated ER-RAC1^P29S^, quantified using RNA-seq (n = 3 independent experiments). (J) Effect of treatment with SRF/MRTF inhibitors on mesenchymal marker induction by ER-RAC1^P29S^. Cells were pre-treated with 5–10 μM CCG-1423 or CCG-203971 1 h before 4OHT treatment (1 μM) for 48 h (n = 3 independent experiments); mRNA levels were determined using qPCR and normalized to *Gapdh* expression. For all graphs: bars represent means ± SD. See also Figure S2.

A reverse-phase protein array was employed to study the cellular signals transmitted by RAC1^P29S^. We found that AKT was increasingly phosphorylated upon ER-RAC1^P29S^ activation in melanocytes (Figures 2A, 2B, and S2A). Conversely, small hairpin RNA (shRNA)-mediated depletion of the endogenous RAC1^P29S^ in a human melanoma cell line suppressed phosphorylation of AKT (Figure S2B). In both of these systems, we found no evidence that RAC1^P29S^ signals via the MAPK pathway (Figures S2B and S2C).

Next, we explored effects of RAC1^P29S^ signaling on the transcriptome of melanocytes. Activation of ER-RAC1^P29S^ induced numerous gene expression changes in melanocytes (Figure 2C). After 4 h, targets of the transcription factor SRF were heavily enriched among the upregulated genes, while no other transcription factor was significantly linked to the gene expression changes (Figures 2D–2F). SRF can be activated via either the ternary complex factor (TCF) or myocardin-related transcription factor (MRTF) co-factor. TCFs are regulated by MAPK signaling, while MRTF is inhibited by monomeric actin and can be activated by RHO GTPases that polymerize actin and thereby relieve the inhibition (Esnault et al., 2014, Miralles et al., 2003). When assessing the relative contribution of TCF versus MRTF signatures in our gene expression data, we found that MRTF signatures were particularly enriched and any enrichment effect of TCF signatures could be attributed to target overlap with MRTF (Figures S2D–S2H).

Forty hours after activating ER-RAC1^P29S^, many potentially secondary and tertiary gene expression changes were observed. Pathway enrichment analysis revealed that genes involved in epithelial-to-mesenchymal transition (EMT) were most strongly enriched among upregulated genes (Figures 2G–2I). EMT can be viewed a misnomer in this context, given that melanocytes are not epithelial-lineage cells. We searched for EMT-driving transcription factors among upregulated genes and indeed found that the SRF/MRTF targets *Snai2* and *Jun* were upregulated, suggesting a link between SRF/MRTF activation and promotion of a mesenchymal-like state (Figure S2I). To establish a causal relationship, we activated ER-RAC1^P29S^ in the presence of the specific SRF/MRTF inhibitors CCG-1423 and CCG-203971 (Haak et al., 2017). This suppressed the induction of *Snai2* and *Jun*, and mesenchymal marker gene and protein expression (Figures 2J, S2J, and S2K), indicating that ER-RAC1^P29S^ initiates a melanocytic to mesenchymal transition through SRF/MRTF. Similar results were obtained when MRTF was depleted using RNAi (Figure S2L). Depletion of the EMT transcription factors Snai2 and Jun significantly reduced the survival ability of melanocytes with activated RAC1^P29S^ (Figure S2M). Similar to results at 4 h, transcription factor target enrichment analysis demonstrated that SRF was the dominant driver of gene expression changes following 40 h of ER-RAC1^P29S^ activation (Figure S2N). To assess the effect of PAK on activation of SRF/MRTF and the transition to a mesenchymal identity, we used the PAK inhibitor G-5555 and quantified gene expression of canonical SRF/MRTF targets and mesenchymal markers. We observed that PAK is not involved in the activation of SRF/MRTF, but does contribute to the transition to a mesenchymal identity (Figure S2O).

Taken together, unbiased proteomic and transcriptomic analyses revealed that melanocytic ER-RAC1^P29S^ activates AKT and initiates a shift toward a more mesenchymal transcriptional state via SRF/MRTF and PAK.

### Endogenous RAC1^P29S^ in Melanocytes Recapitulates the Effects of ER-RAC1^P29S^

**Figure 3:**
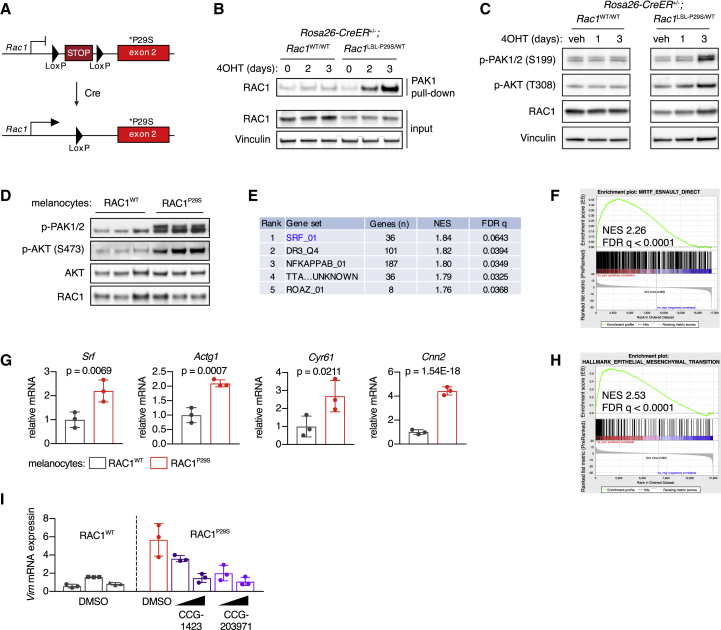
Impact of Endogenous RAC1^P29S^ on AKT and SRF/MRTF Activation in Melanocytes and Mesenchymal Phenotype (A) Schematic of the *Rac1*^LSL−P29S^ allele. (B) *Rosa26-CreER*^+/–^*;Rac1*^LSL−P29S/WT^ and *Rosa26-CreER*^+/–^*;Rac1*^WT/WT^ MEFs were isolated and recombined using 1 μM 4OHT. Activation of RAC1 was assessed using pull-down assay. (C) Phosphorylation of PAK1/2 and AKT in MEFs upon recombination of the *Rac1*^LSL−P29S^ allele by 4OHT treatment (1 μM). (D) Phosphorylation of PAK1/2 and AKT in mouse melanocytes with endogenous RAC1^P29S^. Immunoblot of three independent cultures per genotype is shown. (E) Transcription factor target enrichment analysis of gene expression changes in melanocytes with endogenous RAC1^P29S^ versus RAC1^WT^. GSEA was performed using the transcription factor target gene set collection from MSigDB in combination with RNA-seq data of melanocytes from *Rac1*^LSL−P29S/WT^ mice versus *Rac1*^WT/WT^ mice (n = 3 independent cultures per genotype). (F) GSEA results using SRF/MRTF/TCF gene sets from Esnault et al. (2014), was applied on RNA-seq data from melanocytes cultured with endogenous RAC1^P29S^ versus RAC1^WT^. GSEA plot for direct targets of MRTF is shown. (G) Normalized mRNA read counts of canonical SRF/MRTF targets in melanocytes with endogenous RAC1^P29S^ (n = 3 independent cultures per genotype); Wald test was applied in combination with Benjamini-Hochberg correction. (H) GSEA plot of the curated EMT hallmark gene set from MSigDB using RNA-seq data of melanocytes from *Rac1*^LSL−P29S/WT^ mice versus *Rac1*^WT/WT^ mice (n = 3 independent cultures per genotype). (I) Effect of SRF/MRTF pathway inhibition on mRNA expression of vimentin in mouse melanocytes with endogenous RAC1^P29S^. Cells were treated with 10–25 μM CCG-1423 or CCG-203971 and starved of TPA for 24 h (n = 3 independent experiments). For all graphs: bars represent means ± SD. See also Figure S3.

Conclusions drawn from previous studies regarding the functions of RAC1^P29S^, including those described above, have relied on ectopic expression experiments or the comparison between endogenous RAC1^P29S^ mutant and RAC1^WT^ human melanoma cell lines, which have other genetic alterations. To enable more controlled modeling of the impact of endogenous RAC1^P29S^, we generated mice carrying a conditional P29S mutation targeted to the endogenous *Rac1* locus. A Lox-stop-Lox (LSL) cassette was inserted upstream of a mutated exon 2, which produces a null allele in the absence of Cre recombinase and a P29S mutant allele following exposure to Cre recombinase activity (Figure 3A). Homozygous *Rac1*^LSL−P29S^ was embryonically lethal in mice lacking Cre, in concordance with results from *Rac1* knockout mice (Sugihara et al., 1998) (Figure S3A). We also observed some degree of lethality in heterozygous mice, indicating that two copies of the wild-type *Rac1* allele are required for optimal development (Figure S3B).

When we isolated mouse embryonic fibroblasts (MEFs) from *Rac1*^LSL−P29S/WT^ mice crossed onto a ubiquitously expressed conditional Cre driver (Rosa26-CreER), recombination resulted in increased binding of RAC1 to its effector PAK1 and elevated levels of phospho-PAK and phospho-AKT (Figures 3B, 3C, and S3C). To study the effects of RAC1^P29S^ in a more relevant cellular context, melanocytes were isolated from *Rac1*^LSL−P29S/WT^ mice and *Rac1*^WT/WT^ littermates crossed onto a melanocyte-specific inducible Cre (Tyr-CreER; Figure S3D). As observed in MEFs and in ER-RAC1^P29S^ melanocytes, phosphorylation of PAK and AKT was consistently increased in cells expressing RAC1^P29S^, while phospho-ERK levels were not (Figures 3D and S3E). Three melanocyte cultures expressing endogenous RAC1^P29S^ and three RAC1^WT^ cultures were subjected to mRNA sequencing to investigate effects on gene expression. Targets of SRF/MRTF were most enriched among upregulated genes compared with other transcription factors (Figures 3E–3G). In addition, we detected a strong presence of the EMT gene expression signature and commonly used mesenchymal markers were increased at the mRNA and protein levels (Figures 3H and S3E–S3G). SRF/MRTF inhibition reduced expression of the mesenchymal markers vimentin and N-cadherin in mouse melanocytes with endogenous RAC1^P29S^ (Figures 3I and S3H).

In summary, melanocyte cultures expressing RAC1^P29S^ from the endogenous locus showed increased activation of PAK, AKT, and SRF/MRTF, and displayed a more mesenchymal gene expression profile, confirming our findings made with ectopic expression systems.

### Melanocytes and Melanoma Cells Driven by RAC1^P29S^ Depend on AKT and MRTF for Survival

**Figure 4:**
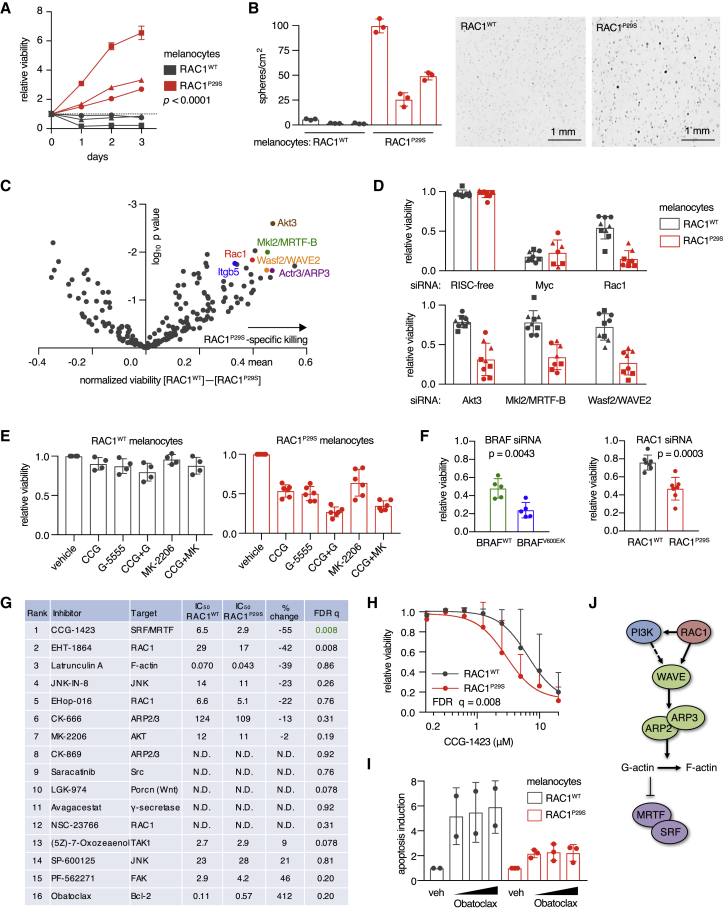
Discovery of AKT and MRTF Dependencies in Cells with Endogenous RAC1^P29S^ (A) Melanocyte cultures from Rac1^WT/WT^ and Rac1^LSL−P29S/WT^ mice were grown in growth factor-reduced medium (FCS 0.25%, TPA-free) and assayed for viability using CellTiter-Glo; p value using two-way ANOVA for the genotype indicated. (B) Single-cell suspensions of melanocytes from *Rac1*^WT/WT^ and *Rac1*^LSL−P29S/WT^ mice (n = 3 independent cultures per genotype) were seeded in soft agar for 3 weeks before imaging and automated counting. (C) Melanocytes from *Rac1*^WT/WT^ and *Rac1*^LSL−P29S/WT^ mice were transfected with a custom siRNA library (n = 205 pools), grown in TPA-free medium for 3 days and assayed for viability (n = 3 independent cultures per genotype, each assayed in three independent experiments). (D) Detailed plots of the data from the RAC1 effector siRNA screen presented in (C). Control siRNAs (top) and siRNAs that specifically kill melanocytes with endogenous RAC1^P29S^ (bottom). (E) Effect of combination treatments with inhibitors of the SRF/MRTF, PAK, and AKT pathways on viability of melanocytes with RAC1^WT^ (left) or endogenous RAC1^P29S^ (right). Mouse melanocyte cultures were grown in regular medium and assayed for viability 72 h after starting drug treatments. Inhibitor doses: CCG-203971 (SRF/MRTF) 10 μM, G-5555 (PAK) and MK-2206 (AKT) 2 μM. (F) Effects on viability of human melanoma cell lines of reduction in expression of RAC1 or BRAF. Cells were cultured in growth medium and assayed for viability at 96 h post-transfection (n = 5 cell lines per genotype for the BRAF panel, n = 7 cell lines per genotype for the RAC1 panel, dots represent means of a single cell line that was tested in multiple independent experiments); p values from t test. (G) Drug panel targeting RAC1 effectors applied in human melanoma cell lines with RAC1^P29S^ versus cell lines with RAC1^WT^. Cells were cultured in growth medium and assayed for viability 72 h after starting drug treatments. Drugs ranked by the percentage difference between the half maximal inhibitory concentration (μM) of RAC1^WT^ cell lines and RAC1^P29S^ cell lines. For each drug, two-way ANOVA was used to probe the full dataset for a genotype effect, which was corrected according to Benjamini-Hochberg to produce FDR q values; N.D., not determined because curve fitting failed. (H) Viability of human melanoma cell lines treated with the SRF/MRTF inhibitor CCG-1423 was determined using CellTiter-Glo (n = 7 cell lines per genotype, for each cell line the mean from two independent experiments was used); statistical analysis was performed as described in (F). (I) Apoptosis of mouse melanocytes with endogenous RAC1^P29S^ or RAC1^WT^ cultured in growth medium and treated with obatoclax at 250 nM, 500 nM, or 1 μM for 3 days (n = 2–3 independent cultures per genotype). (J) Schematic of the SRF/MRTF signaling pathway. For all graphs: bars represent means ± SD. See also Figure S4.

Long-term culture of melanocytes in full medium demonstrated that RAC1^P29S^ does not alter their rate of proliferation or immortalization (Figure S4A). However, endogenous RAC1^P29S^ promoted survival when melanocytes were cultured in the absence of growth factors or seeded at very low densities (Figures 4A and S4B). Moreover, we observed increased sphere-forming ability when the cells were seeded in soft agar (Figure 4B) and protection against apoptosis when cells were starved of growth factors and adhesion (Figure S4C).

We interrogated which downstream effectors are used by RAC1^P29S^ for the promotion of survival in the hope of uncovering therapeutic targets for P29S mutant RAC1-driven melanoma. To study this in a systematic manner, we designed a RAC1 effector RNAi screen. We included genes whose products are known to directly bind RAC proteins (gene ontology GO:0048365, n = 31); selected genes whose products are reported to function in signaling pathways downstream of RAC1 activation (PAK, MAPK, PI3K, SRF, NF-κB, JNK, STAT3, Wnt, and integrin signaling; n = 53); and genes upregulated in ER-RAC1^P29S^ melanocytes that are associated with cell survival, plus the transcription factors suspected to be driving these gene expression changes (n = 115). Three melanocyte cultures from each *Rac1* genotype were transfected with the arrayed custom small interfering RNA (siRNA) library, grown in growth factor-reduced medium and assayed for viability 96 h after transfection. Effective knockdown was suggested by near-complete killing efficiency for multiple siRNA pools, including Myc, and experimentally verified for the Mkl2 pool (Figure S4D). We particularly focused on genes that, when depleted, specifically reduced viability of RAC1^P29S^ melanocytes compared with RAC1^WT^ counterparts. An siRNA pool targeting RAC1 itself was included as a control and was among the top hits in the screen, validating our experimental system (Figures 4C and 4D). This approach identified Akt3 and components of the WAVE→ARP2/3→SRF/MRTF pathway to be specifically required for survival in RAC1^P29S^ melanocytes. These findings were confirmed using small molecules targeting AKT, SRF/MRTF, and PAK signaling (Figure 4E). We observed that inhibition of these pathways had a cumulative effect on viability, suggesting that SRF/MRTF promotes survival downstream of RAC1^P29S^ by a mechanism distinct from either AKT or PAK. Interestingly, the flatter and less dendritic morphology of melanocytes with endogenous RAC1^P29S^ could be partially reversed with an SRF/MRTF inhibitor (Figure S4E). Of note, depletion of EMT transcription factors inhibited survival of melanocytes with endogenous RAC1^P29S^ (Figure S4F), similar to effects observed in melanocytes with inducible ER-RAC1^P29S^.

There are insufficient RAC1^P29S^ cases in public clinical datasets to permit robust statistical analyses (e.g., *RAC1*^P29S^ is present in 2/1,020 and 11/366 cases in the Cancer Cell Line Encyclopedia and The Cancer Genome Atlas [TCGA] melanoma cohort, respectively), so we turned to whole-genome shRNA screening data from human cancer cell lines (Tsherniak et al., 2017), which were used to determine co-dependencies in human cancer cells that require RAC1 for viability. This analysis demonstrated that RAC1-dependent cancer cells are particularly sensitive to depletion of WAVE complex subunits, ARP2/3 subunits, and focal adhesion components (Figure S4G). These results were confirmed in an independent dataset that was established using a smaller but more specific shRNA library (McDonald et al., 2017) (Figure S4H). Next, we assessed a selection of chemical inhibitors in *RAC1*^P29S^ human melanoma cells in an attempt to validate any possible vulnerabilities uncovered by our earlier studies. Chemical inhibitors may also help bypass problems of isoform redundancy. We assembled a panel consisting of seven melanoma cell lines with endogenous *RAC1*^P29S^ and seven control melanoma cell lines with *RAC1*^WT^ (Figure S4I). First, RAC1 was knocked down to determine its contribution to the survival of *RAC1*^P29S^ human melanoma cells. Knockdown was more harmful to *RAC1*^P29S^ cell lines compared with *RAC1*^WT^ cell lines, in a similar fashion to the differential effect of BRAF knockdown in *BRAF* mutants (Figure 4F). Subsequently, we selected 16 inhibitors targeting candidate RAC1 effector pathways and obtained dose-response viability curves (Figure 4G). Although this approach is inherently “noisy” due to the significant genetic heterogeneity across human melanoma cell lines, we were nonetheless able to detect a statistically significant sensitivity of *RAC1*^P29S^ cell lines to the SRF/MRTF inhibitor CCG-1423, and to the RAC1 activation inhibitor EHT-1864 (Figure 4H). Interestingly, *RAC1*^P29S^ cells tend to be more resistant to the anti-apoptotic Bcl-2 family inhibitor obatoclax, suggesting that *RAC1*^P29S^ cells are generally more resistant to apoptosis (Figure S4J). When we applied obatoclax and another Bcl-2 family inhibitor, navitoclax, in mouse melanocytes, RAC1^P29S^ protected against induction of apoptosis (Figures 4I and S4K). This effect appeared specific to inhibition of the Bcl-2 family anti-apoptotic pathway, as RAC1^P29S^ did not suppress apoptosis induced by chemotherapeutic agents topotecan and etoposide (Figure S4K). Finally, we tested whether *RAC1*^P29S^-specific protection of apoptosis was mediated through PAK, AKT, or SRF/MRTF. Combined chemical inhibition of the SRF/MRTF and AKT pathways negated protection of obatoclax-induced apoptosis (Figure S4L).

Taken together, these data suggest that RAC1^P29S^ activates SRF/MRTF via WAVE and ARP2/3, and also activates AKT, to suppress apoptosis and promote survival of melanocytes and melanoma cells (Figure 4J).

### RAC1^P29S^ Promotes Tumorigenesis when Expressed in the Whole Body

**Figure 5:**
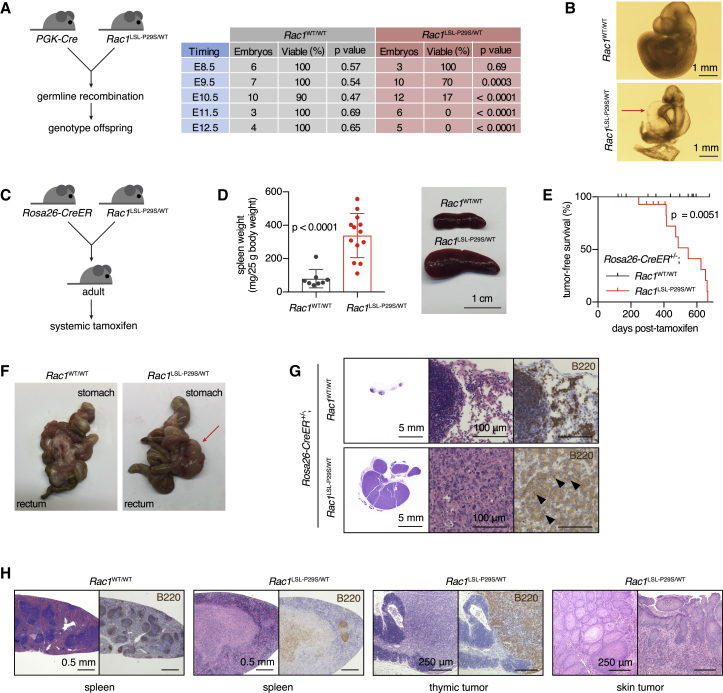
Effect of Ubiquitous Expression of RAC1^P29S^*In Vivo* on Induction of B Cell Lymphoma (A) Schematic of *PGK-Cre/Rac1*^LSL−P29S^ cross and summary of results from the timed analysis of embryos. *PGK-Cre* was always maternal to ensure germline Cre recombination in all embryos. p values derived from chi-square testing are indicated. (B) Representative micrographs showing gross cardiovascular abnormalities and developmental retardation in *Rac1*^LSL−P29S/WT^ embryos (E10.5). Red arrow, enlarged pericardial cavity. (C) Schematic of experimental design to express RAC1^P29S^ in the whole body of adult mice. Tamoxifen was administered by oral gavage. (D) Representative image of the spleen and quantification of spleen weight in aged mice with the indicated genotype. Bars represent means ± SD (n = 8–13 mice per genotype); Mann-Whitney test used for statistical comparison. (E) Tumor-free survival curve of mice with indicated genotypes after treatment with tamoxifen. Result from log rank testing (Mantel-Cox) is indicated. (F) Representative photos of the digestive tract from a *Rosa26-CreER*^+/–^*;Rac1*^WT/WT^ mouse and a mesenteric lymphoma-bearing *Rosa26-CreER*^+/–^*;Rac1*^LSL−P29S/WT^ mouse. Red arrow, lymphoma. (G) Representative H&E- and B220-stained sections of a mesenteric lymphoma versus normal mesenteric lymph nodes in a control mouse. (H) Representative H&E- and B220-stained sections of a normal spleen from a *Rosa26-CreER*^+/–^*;Rac1*^WT/WT^ mouse and the spleen, thymic lymphoma, and squamous cell tumor of the skin from *Rosa26-CreER*^+/–^*;Rac1*^LSL−P29S/WT^ mice. See also Figure S5.

We began our investigations of the *in vivo* effects of RAC1^P29S^ by inducing its expression in the germline of mice. Crossing *Rac1*^LSL−P29S/WT^ males and *PGK-Cre* females with oocytic Cre expression only produced live offspring with a *Rac1*^WT/WT^ genotype (Figure S5A). Timed analysis revealed that embryos with germline *Rac1*^P29S/WT^ displayed general developmental delay, gross enlargement of the pericardial cavity, and embryonic lethality 10.5 days post-fertilization (Figures 5A and 5B).

To circumvent embryonic lethality, we generated *Rosa26-CreER*^+/–^*;Rac1*^LSL−P29S/WT^ mice and recombined *Rac1*^LSL−P29S^ in the whole body of adults by systemic tamoxifen administration (Figures 5C, S5B, and S5C). Our first clear observation was that *Rac1*^LSL−P29S/WT^ mice were smaller compared with *Rac1*^WT/WT^ littermates, which was dependent on tamoxifen-mediated recombination (Figures S5D and S5E). After aging animals for 1–2 years, *Rac1*^LSL−P29S/WT^ mice developed splenomegaly and tumors, while wild-type controls did not (Figures 5D and 5E). The tumors were mostly mesenteric lymphomas, occasionally accompanied by a thymic lymphoma and/or a squamous cell tumor of the skin (Figures 5F–5H and S5F). Histological and flow cytometric characterization of the mesenteric lymphomas demonstrated that the B cell compartment was transformed, whereas the T cell compartment appeared normal (Figures 5G, S5G, and S5H).

Thus, ubiquitous expression of RAC1^P29S^ at endogenous levels in adult mice promotes gradual development of B cell lymphoma, while being developmentally lethal when expressed in the embryo.

### RAC1^P29S^ Promotes Melanomagenesis

We did not observe melanomas in mice with RAC1^P29S^ expression in the whole body, so we proceeded to use the *Tyr-CreER* mouse model with topical 4OHT treatment to express RAC1^P29S^ exclusively in melanocytes in combination with other genetic aberrations commonly found in melanoma (Dankort et al., 2009, Dhomen et al., 2009). First, we mined public sequencing databases to uncover the genomic landscape of *RAC1*^*P29S*^ human cutaneous melanoma. By compiling data from multiple sources, we identified a substantial number of cases carrying *RAC1*^*P29S*^ (n = 51). In addition, we identified a smaller number of cases with a *RAC1*^*P29L*^ mutation (n = 5), which is also thought to be activating (Kawazu et al., 2013, Alan and Lundquist, 2014). In 82% of *RAC1*^*P29S/L*^ cases analyzed there was a co-occurring mutation in one of the three main melanoma driver genes *BRAF*, *NRAS*, and *NF1* (Figure S6A). Rates of *BRAF* and *NRAS* hotspot mutations were similar in *RAC1*^*P29S/L*^ melanomas compared with melanoma in general, while co-occurrence of *NF1*-inactivating mutations and *RAC1*^*P29S/L*^ was significantly enriched in two large melanoma cohorts (cBioPortal cohort, n = 709, log odds ratio 1.73, p < 0.001; GENIE cohort, n = 1,868, log odds ratio 1.71, p < 0.001). It should be noted that these observations could be influenced by the fact that both *RAC1*^*P29S/L*^ and *NF1* mutations tend to occur preferentially in highly mutated melanomas carrying a UV mutational signature (Cancer Genome Atlas Research Network, 2014).

**Figure 6:**
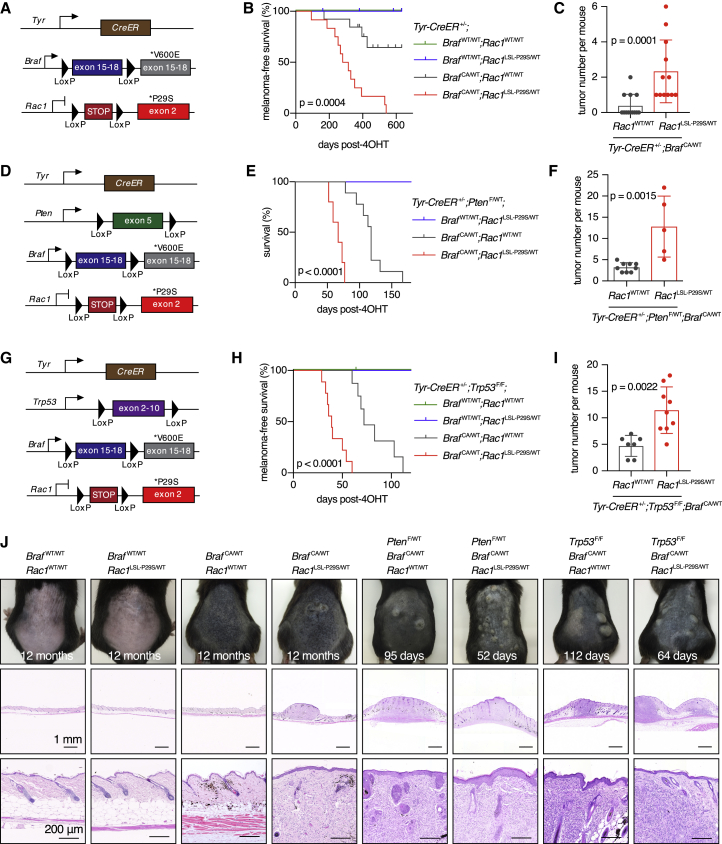
Effect of Melanocytic Expression of RAC1^P29S^*In Vivo* on Tumorigenesis (A) Schematic of the *Tyr-CreER*, *Braf*^CA^ and *Rac1*^LSL−P29S^ allele combination. (B) Melanoma-free survival curves of *Tyr-CreER*^+/–^*;Braf*^CA/WT^*;Rac1*^WT/WT^ mice and *Tyr-CreER*^+/–^*;Braf*^CA/WT^*;Rac1*^LSL−P29S/WT^ mice were compared using log rank testing (Mantel-Cox). (C) Effects of RAC1^P29S^ on tumor number in *Tyr-CreER*^+/–^*;Braf*^CA/WT^ mice. Groups were compared using the Mann-Whitney test (n = 12–13 mice per genotype). (D) Schematic of the *Tyr-CreER*, *Pten*^F^, *Braf*^CA^, and *Rac1*^LSL-P29S^ allele combination. (E) Overall survival curves of *Tyr-CreER*^+/–^*;Pten*^F/WT^*;Braf*^CA/WT^*;Rac1*^WT/WT^ mice and *Tyr-CreER*^+/–^*;Pten*^F/WT^*;Braf*^CA/WT^*;Rac1*^LSL−P29S/WT^ mice were compared using the log rank testing (Mantel-Cox). (F) Effects of RAC1^P29S^ on tumor number in *Tyr-CreER*^+/–^*;Pten*^F/WT^*;Braf*^CA/WT^ mice. Groups compared using Mann-Whitney testing (n = 5–9 mice per genotype). (G) Schematic of the *Tyr-CreER*, *Trp53*^F^, *Braf*^CA^, and *Rac1*^LSL−P29S^ allele combination. (H) Melanoma-free survival curves of *Tyr-CreER*^+/–^*;Trp53*^F/F^*;Braf*^CA/WT^*;Rac1*^WT/WT^ mice and *Tyr-CreER*^+/–^*;Trp53*^F/F^*;Braf*^CA/WT^*;Rac1*^LSL−P29S/WT^ mice were compared using log rank testing (Mantel-Cox). (I) Effects of RAC1^P29S^ on tumor number in *Tyr-CreER*^+/–^*;Trp53*^F/F^*;Braf*^CA/WT^ mice. Groups compared using an unpaired two-tailed t test (n = 7–9 mice per genotype). (J) Representative photographs and histology of the dorsal skin of various mouse melanoma models. All mice are *Tyr-CreER*^+/–^, with additional alleles indicated. Time since 4OHT treatment is indicated. For all graphs: bars represent means ± SD. See also Figure S6.

When we introduced RAC1^P29S^ alone in melanocytes, mice did not develop melanocytic hyperplasia or melanoma (n = 11 mice, aged for an average of 540 days; Figures 6A and 6B). In contrast, BRAF^V600E^ alone produced melanocytic hyperplasia, which in 4 out of 13 mice progressed to melanoma (aged for an average of 461 days; Figure 6B). Strikingly, mice with combined BRAF^V600E^ and RAC1^P29S^ always developed melanoma and the number of tumors per mouse was significantly increased (n = 12 mice, aged for an average of 394 days; Figure 6C). The tumor-promoting effect of RAC1^P29S^ was also clearly observed in BRAF^V600E^;*Pten*-hemizygous (Figures 6D–6F) and BRAF^V600E^;*Trp53*-null melanoma models (Figures 6G–6J). Recombination of the *Rac1*^LSL−P29S^ allele in tumors was confirmed at both the DNA and mRNA level (Figures S6B and S6C). In the BRAF^V600E^;*Pten*-hemizygous melanoma model we did not see obvious evidence for loss of the second *Pten* copy, either with or without the *Rac1*^LSL−P29S^ allele.

We also evaluated the effect of RAC1^P29S^ in the aggressive BRAF^V600E^;*Pten*-null melanoma model (Dankort et al., 2009). RAC1^P29S^ did not affect melanoma formation in this setting, likely due to the already very rapid disease progression in BRAF^V600E^;*Pten*-null;RAC1^WT^ animals (Figures S6D–S6F). Because *RAC1*^P29S/L^- and *NF1*-inactivating mutations co-occur in human melanoma, we also elected to cross the *Rac1*^LSL−P29S^ allele onto a *Nf1*-null;*Trp53*-null melanoma model, which has not been reported before. In this context, RAC1^P29S^ cooperated with loss of NF1 and p53 to promote melanomagenesis (Figures S6G–S6I).

We investigated our two most aggressive mouse melanoma models to determine if RAC1^P29S^ promotes spontaneous lung metastasis. Analysis of lungs from both the BRAF^V600E^;*Trp53*-null model and the BRAF^V600E^;*Pten*-null model revealed a low number of lung nodules in *Rac1*^WT/WT^ mice, which were in part benign primary bronchoalveolar tumors and in part genuine melanoma metastases (Figures S6J–S6N). The number of lung nodules was not significantly increased in *Rac1*^LSL−P29S/WT^ mice, indicating that RAC1^P29S^ does not promote metastasis in these models, despite the well-known effects of RAC1 signaling on cell motility *in vitro* (Marei and Malliri, 2017).

In summary, we used BRAF^V600E^- and NF1-loss-driven melanoma models to demonstrate that endogenous RAC1^P29S^ promotes the progression of melanocytic hyperplasia to melanoma. This effect is also seen in the context of loss of PTEN and p53 tumor suppressors.

### RAC1^P29S^ Promotes Mesenchymal Differentiation in Melanomas and Resistance to BRAF Inhibition

To gain a clearer molecular understanding of the effect of RAC1^P29S^ on the development of primary melanomas we used the BRAF^V600E^;*Pten*-hemizygous model because of the clear phenotype and access to sufficient RAC1^WT^ control tumors. Because RAC1^P29S^ promotes the progression of BRAF^V600E^-induced melanocytic hyperplasia to melanoma, we considered that RAC1^P29S^ may achieve this by suppressing oncogene-induced senescence. However, we could not find clear support for this hypothesis when evaluating normal skin and tumors for senescence markers and gene expression signatures (Figures S7A–S7D).

**Figure 7:**
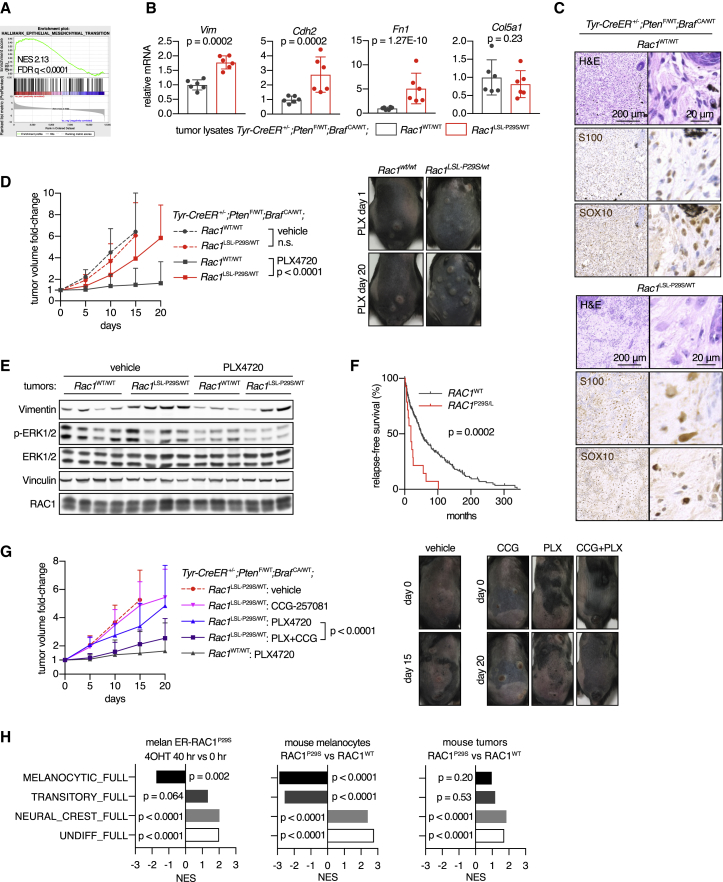
Effect of RAC1^P29S^ Drug Resistance and Mesenchymal Phenotype in BRAF-Driven Melanoma (A) GSEA plot of the EMT hallmark gene set from MSigDB, using RNA-seq data of tumor lysates from *Tyr-CreER*^*+/–*^*;Pten*^F/WT^*;Braf*^CA/WT^*;Rac1*^LSL−P29S/WT^ mice versus tumor lysates from *Tyr-CreER*^+/–^*;Pten*^F/WT^*;Braf*^CA/WT^*;Rac1*^WT/WT^ mice (n = 6 tumors from five to six animals per group). (B) Normalized mRNA read counts of mesenchymal markers in tumor lysates from *Tyr-CreER*^+/–^*;Pten*^F/WT^*;Braf*^CA/WT^ mice. For statistical comparison of groups, Wald test was applied in combination with Benjamini-Hochberg correction (n = 6 tumors from five to six animals per genotype). (C) Effect of expression of RAC1^P29S^ in melanoma on tumor architecture. Representative tumor sections shown stained with H&E or melanoma cell markers S100 and SOX10 (immunohistochemistry). (D) Tumors were induced in *Tyr-CreER*^+/–^*;Pten*^F/WT^*;Braf*^CA/WT^ mice by topical 4OHT. After tumors were established, animals were treated with PLX4720 incorporated in chow. Tumor growth curves were compared using two-way ANOVA, with the p value for the genotype effect indicated (n = 18–44 tumors from 6 to 10 animals per data point). Representative photos of tumors are included. (E) Tumors at the end of the experiment presented in (D) were harvested, lysed and used for immunoblotting with indicated antibodies. (F) Influence of *RAC1*^P29S/L^ mutation status on relapse-free survival in patients with melanoma. Data from the TCGA cutaneous melanoma cohort (n = 309 patients, 45% *BRAF*^mut^ and 55% *BRAF*^WT^) were used. Groups were compared using log rank testing (Mantel-Cox), with the p value indicated. (G) After tumors were established by topical 4OHT, animals were treated with PLX4720 incorporated in chow and CCG-257081 by oral gavage. Tumor growth curves were compared using two-way ANOVA, with the p value for the genotype effect indicated (n = 10–15 tumors from 3 to 4 animals per data point). (H) GSEA was performed using the four melanoma differentiation state signatures developed by Tsoi et al. (2018), in combination with RNA-seq dataset of changes in ER-RAC1^P29S^ melanocyte-treated 4OHT (n = 3 independent experiments; left panel), RNA-seq data from melanocytes with endogenous RAC1^P29S^ versus RAC1^WT^ (n = 3 independent cultures per genotype; middle panel) and RNA-seq data of tumor lysates from *Tyr-CreER*^+/–^*;Pten*^F/WT^*;Braf*^CA/WT^*;Rac1*^LSL−P29S/WT^ mice versus *Tyr-CreER*^+/–^*;Pten*^F/WT^*;Braf*^CA/WT^*;Rac1*^WT/WT^ mice (n = 6 tumors from five to six animals per group; right panel); p values are indicated. For all graphs: means ± SD is shown. See also Figure S7.

Pathway enrichment analysis of mRNA sequencing data showed the presence of SRF/MRTF and mesenchymal signatures in RAC1^P29S^ tumors compared with RAC1^WT^ tumors (Figures 7A, 7B, S7E, and S7F). *In situ* hybridization demonstrated increased mRNA expression of two canonical SRF/MRTF targets in tumor cells, substantiating the finding of increased SRF/MRTF signaling in the RAC1^P29S^ melanoma (Figure S7G). Histologically, the RAC1^WT^ lesions had a relatively uniform cellularity with predominant spindle cells and more compact epithelioid rounded cells in the upper half of the dermis. In contrast, the RAC1^P29S^ lesions had a neurotized appearance, containing larger, elongated tumor cells (Figure 7C). An extracellular matrix gene expression signature was found to be highly enriched in RAC1^P29S^ tumors compared with RAC1^WT^ tumors, being the most significant of the 4,838 gene sets in the curated C2 database (Broad) (Figure S7H).

First-line therapy of patients with BRAF mutant melanoma consists of treatment with a BRAF kinase inhibitor, which often produces a good initial response before resistance to therapy rapidly emerges. We studied BRAF inhibitor responses using the BRAF^V600E^;*Pten*-hemizygous melanoma model. RAC1^WT^ and RAC1^P29S^ tumor growth rates were similar in vehicle-treated animals, confirming that RAC1^P29S^ does not influence tumor cell proliferation (Figure 7D). In contrast, the RAC1 genotype had a significant effect when animals were treated with the BRAF inhibitor PLX4720. Although RAC1^WT^ tumors were generally stable or regressed, RAC1^P29S^ tumors were clearly able to grow under PLX4720 therapy. In line with *in vitro* data, we did not detect activation of the MAPK pathway in mutant tumors exposed to therapy (Figure 7E). Both untreated and inhibitor-treated mutant tumors had higher expression of the mesenchymal marker vimentin (Figures 7E and S7I). In the TCGA melanoma cohort, with or without *BRAF*^mut^, *RAC1*^P29S/L^ mutation, or *RAC1* overexpression is also associated with relapse (Figures 7F and S7J). Mouse melanoma cell cultures with RAC1^P29S^ showed lower levels of apoptosis when exposed to a BRAF inhibitor (Figure S7K), supporting the idea that the resistance to therapy observed in our mouse model resulted from RAC1^P29S^-mediated suppression of apoptosis. In an attempt to reverse resistance to BRAF inhibition, we applied the optimized SRF/MRTF inhibitor CCG-257081 (Hutchings et al., 2017). Co-treatment with CCG-257081 significantly suppressed tumor growth in PLX4720-treated mice (Figure 7G).

Finally, when we map the impact of RAC1^P29S^ on transcriptional data from our various *in vitro* and *in vivo* model systems against four recently defined, sequential melanoma differentiation states (Tsoi et al., 2018), we see that RAC1^P29S^ induces de-differentiation from a melanocytic toward an undifferentiated state, which has been associated with increased resistance to BRAF inhibitors (Figure 7H).

In summary, endogenous expression of RAC1^P29S^ in BRAF-driven melanoma produces a less melanocytic and more mesenchymal differentiation state and generates resistance to the BRAF inhibitor PLX4720, which can be substantially reversed by co-treatment with an SRF/MRTF inhibitor.

## Discussion

We have investigated the impact of signaling by RAC1, an oncogene which is mutationally activated in 4% of melanomas and may be activated by alterations in upstream regulators in a larger fraction, showing that RAC1^P29S^ promotes the initiation of melanoma driven by mutant BRAF or NF1 loss. Systematic analysis of effectors reveals that endogenous expression of RAC1^P29S^ activates PAK, AKT, and also a WAVE→ARP2/3→SRF/MRTF cascade, which induces switching from a melanocytic to a mesenchymal-like cellular identity. The end result is that RAC1^P29S^ cells have enhanced tumorigenic and survival abilities due to suppression of apoptosis, with the important consequence that resistance to BRAF inhibitors is generated. RAC1^P29S^ does not appear to promote melanoma formation and progression through opposing cellular senescence or by enhancing the metastatic process.

Our data suggest a model where RAC1^P29S^ signaling triggers a transcriptional program that has several effects in melanoma cells, including suppression of melanocytic differentiation, induction of a mesenchymal-like phenotype, and increased survival and resistance to therapy. Recently, transcriptional analysis has been used to define four different interlinked differentiation states for melanoma subtypes with differential resistance to drugs, such as BRAF inhibitors, and vulnerability to oxidative stress (Tsoi et al., 2018). In this analysis, selective pressure with a BRAF inhibitor promotes transition from a melanocytic/differentiated state through a transitory state to a neural crest state, and finally an undifferentiated state. The latter states are most resistant to BRAF inhibition, but also show increased sensitivity to oxidative stress. When we map the transcriptional data from our different models, both *in vitro* and *in vivo*, we see clearly that RAC1^P29S^ promotes the adoption of the most undifferentiated and least melanocytic states, which are associated with BRAF inhibitor resistance. It is also interesting to speculate whether the known ability of RAC signaling to promote oxidative stress via its control of NADPH oxidase (Ogrunc et al., 2014) might suggest that RAC pathway activation could be involved in the increased vulnerability to oxidative stress of the most undifferentiated melanoma subtypes observed by Tsoi et al. (2018).

Our results suggest that RAC1 signaling promotes a phenotypic switch mediated in part by SRF/MRTF to a mesenchymal differentiation state that is characterized by resistance to apoptosis induction, possibly mediated through control of the Bcl-2 family of apoptosis regulators. Investigation of the expression level of Bcl-2 family proteins in response to RAC1 activation does not indicate that this control is likely to be attributable to a single member, but rather an aggregate of gene expression changes across this family and possibly other apoptotic regulators as well. Because RAC1^P29S^ induces dedifferentiation, it may be that part of its tumor-promoting potential lies in generating more progenitor-like cells from which a tumor can be established, and in making these cells less sensitive to cell death by apoptosis, whether this is induced by a deficient microenvironment or therapy with BRAF inhibitor targeting the main driver oncogene.

Our findings here suggest that the RAC1^P29S^ genotype may identify patients who are less likely to benefit from BRAF inhibitors. However, the observation that RAC1^P29S^-driven resistance to BRAF inhibition can be reversed by inhibiting the SRF/MRTF transcription factor complex provides a possible approach to addressing BRAF inhibitor resistance in the clinic. A series of SRF/MRTF inhibitors have been developed (Hayashi et al., 2014, Lundquist et al., 2014) and used extensively in preclinical models, principally to inhibit fibrosis (Hutchings et al., 2017, Yu-Wai-Man et al., 2017). The findings reported here suggest that SRF/MRTF inhibitors, in combination with BRAF inhibitors, could have utility in the treatment of BRAF mutant melanoma with an additional RAC1^P29S^ mutation. SRF/MRTF inhibitors could present an attractive approach to tackling BRAF inhibitor resistance in melanoma caused by RAC1^P29S^ compared with the use of RAC inhibitors, which have to date proved difficult to progress into the clinic (Marei and Malliri, 2017).

Beyond the 4% of melanoma cases with mutant RAC1, it is also worth speculating whether RAC signaling pathways may be activated in melanoma and perhaps other tumor types by different mechanisms, such as alterations in upstream regulatory proteins such as PREX and TIAM1. If this were the case, then MRTF pathway inhibition might have a broader potential utility in countering BRAF inhibitor resistance, and possibly also resistance to other therapeutic agents. RAC1 function is also known to be essential in KRAS induced tumor formation (Kissil et al., 2007), and in KRAS oncogene addiction (Yuan et al., 2018), so the potential exists for SRF/MRTF inhibition to be explored in a far greater range of tumors than just RAC1^P29S^ melanoma.

## STAR★Methods

### Key Resources Table

**Table:** 

REAGENT or RESOURCE	SOURCE	IDENTIFIER
**Antibodies**		

phospho-AKT (T308)	CST	#13038
phospho-AKT (S473)	CST	#9271
phospho-AKT (S473)	CST	#4060
AKT	CST	#2920
phospho-ERK1/2 (T202/T204)	CST	#9101
ERK1/2	CST	#9107
phospho-PAK1/2 (S199/192)	CST	#2605
phospho-PAK1/2 (T423/402)	CST	#2601
PAK1/2/3	CST	#2604
phospho-MEK1/2 (S217/221)	CST	#9145
RAC1 (23A8)	Merck Millipore	05-389
Vinculin (hVIN-1)	Sigma-Aldrich	V9131
Myc (9E10)	In-house (CRUK LRI)	N/A
Vimentin (EPR3776)	Abcam	ab92547
N-cadherin	CST	#13116
c-Jun	CST	#9165
phospho-c-Jun (S73)	CST	#9164
ER (MC-20)	Santa Cruz	sc-542
Fibronectin	Abcam	ab2413
phospho-BRAF (S445)	CST	#2696
phospho-CRAF (S338)	CST	#9427
S100 (4C4.9)	Abcam	ab4066
SOX10 (A2)	Santa Cruz	sc-365692
p16 (EPR1473)	Abcam	ab108349
p27 (F-8)	Santa Cruz	sc-1641
B220 (RA3-6B2)	BD Biosciences	553086
B220-BV510	Biolegend	RA3-6B2
CD19-BV605	Biolegend	6D5
CD38-APC	Biolegend	90
CD95-BV421	BD Biosciences	Jo2
CD138-BV786	BD Biosciences	281-2
NK1.1-PE	Biolegend	PK136
CD44-APC	Ebioscience	IM7
CD62L-PeCy7	Ebioscience	MEL-14
CD3e-PerCPEFluor710	Ebioscience	145-2C11
CD4-BV421	Biolegend	GK1.5
CD8a-FITC	Ebioscience	53-6.7
PTEN	CST	#9559

**Chemicals, Peptides, and Recombinant Proteins**		

4OH-tamoxifen	Sigma-Aldrich	H6278
Tamoxifen	Sigma-Aldrich	T5648
CCG-1423	Selleckchem	S7719
CCG-203971	Tocris	5277
CCG-257081	In-house (MSU)	N/A
G-5555	Medchem Express	HY-19635
EHT-1864	Tocris	3872
Latrunculin A	Tocris	3973
JNK-IN-8	Selleckchem	S4901
EHop-016	Selleckchem	S7319
CK-666	Sigma-Aldrich	SML0006
MK-2206	Selleckchem	S1078
CK-869	Sigma-Aldrich	C9124
Saracatinib	Cambridge Bioscience	CAY11497
LGK-974	Selleckchem	S7143
Avagacestat	Selleckchem	S1262
NSC-23766	Tocris	2161
(5Z)-7-Oxozeaenol	Tocris	3604
SP-600125	Selleckchem	S1460
PF-562271	Abcam	ab141360
Obatoclax	Selleckchem	S1057
Navitoclax (ABT263)	Selleckchem	S1001
Topotecan	Axxora	4100
Etoposide	Calbiochem	341205
PLX4720	Plexxicon	N.A.
PLX4032/vemurafenib	Selleckchem	S1267

**Critical Commercial Assays**		

Rac1/Cdc42 Activation Assay Kit	Merck Millipore	17-441
RNeasy Mini Kit	Qiagen	74104
DNeasy Blood & Tissue Kit	Qiagen	69506
RNAscope 2.5 LS Reagent Kit	ACDBio	322150

**Deposited Data**		

RNAseq - mouse melanocytes with ER-RAC1^P29S^	GEO repository	GSE118349
RNAseq - mouse melanocytes with endogenous RAC1^P29S^	GEO repository	GSE118343
RNAseq - mouse melanoma with endogenous RAC1^P29S^	GEO repository	GSE118344

**Experimental Models: Cell Lines**		

SK-MEL-2	NCI-60 collection	N/A
SK-MEL-5	NCI-60 collection	N/A
LOX-IMVI	NCI-60 collection	N/A
A2058	Francis Crick Institute	N/A
Colo-792	ECACC	N/A
C32	ECACC	N/A
A375	ATCC	CRL-1619
WM3060	Coriell Institute	WC00126
WM1791C	Coriell Institute	WC00086
IGR-1	DSMZ	ACC-236
YUHEF	Ruth Halaban (Yale)	N/A
YUSOC	Ruth Halaban (Yale)	N/A
YURIF	Ruth Halaban (Yale)	N/A
YUTOGS	Ruth Halaban (Yale)	N/A
293T	Francis Crick Institute	N/A
Phoenix-ECO	Francis Crick Institute	N/A
MCF10A-ECO	Francis Crick Institute	N/A
melan-a	Dorothy Bennett (St. George's)	N/A
XB2 variant	Dorothy Bennett (St George's)	N/A

**Experimental Models: Organisms/Strains**		

*Rac1*^LSL-P29S^ strain	Julian Downward	Rac1^tm1Jdo^
*PGK-Cre* strain (Lallemand et al., 1998)	Francis Crick Institute	Tg(Pgk1-cre)1Lni
*Rosa26-CreER* strain (Seibler et al., 2003)	Taconic	Gt(ROSA)26Sor^tm9(cre/ESR1)Arte^
*Tyr-CreER* strain (Bosenberg et al., 2006)	Jackson Laboratory	Tg(Tyr-cre/ERT2)13Bos
*Braf*^CA^ strain (Dankort et al., 2007)	Jackson Laboratory	Braf^tm1Mmcm^
*Nf1*^F^ strain (Zhu et al., 2001)	NCIMR	Nf1^tm1Par^
*Pten*^F^ strain (Lesche et al., 2002)	Jackson Laboratory	Pten^tm1Hwu^
*Trp53*^F^ strain (Marino et al., 2000)	Axel Behrens	Trp53^tm1Brn^

**Oligonucleotides**		

pLKO.1-sh-scramble: CCGGGCGCGAT AGCGCTAATAATTTCTCGAGAAATTAT TAGCGCTATCGCGCTTTTT	Sigma-Aldrich	SHC016
pLKO.1-sh-hRAC1 no. 70: CCGGGCTAA GGAGATTGGTGCTGTACTCGAGTACAG CACCAATCTCCTTAGCTTTTT	Sigma-Aldrich	TRCN0000004870
pLKO.1-sh-hRAC1 no. 71: CCGGCGCAAAC AGATGTGTTCTTAACTCGAGTTAAGAACAC ATCTGTTTGCGTTTTT	Sigma-Aldrich	TRCN0000004871
GCACCGGCAGGCAGTAACAGTTCA	N/A	Rac1-FP5
GTCGTGTAACTGATGAGCAGGCAGGT	N/A	Rac1-RP5

**Recombinant DNA**		

psPAX2	Paola Scaffidi (Francis Crick Institute)	N/A
pMD2.G	Paola Scaffidi (Francis Crick Institute)	N/A
pcDNA3-2xMyc-HRAS-G12V	cDNA Resource Center (Missouri)	RASH00MNC0
pLZRS-ER-HRASG12V	Paul Khavari (Stanford)	Addgene
pBABE-hygro-empty	ICRF	N/A
pBABE-hygro-2xMyc-Rac1-WT	In-house	clone 8
pBABE-hygro-2xMyc-Rac1-P29S	In-house	clone 1
pBABE-hygro-2xMyc-Rac1-Q61L	In-house	clone 13
pLZRS-ER-Rac1-P29S-shortlinker	In-house	clone 3
pLZRS-ER-Rac1-Q61L-shortlinker	In-house	clone 8

### Contact for Reagent and Resource Sharing

Further information and requests for resources and reagents should be directed to and will be fulfilled by the Lead Contact, Julian Downward (Julian.Downward@crick.ac.uk).

### Experimental Model and Subject Details

#### Transgenic Mice

All animal experiments were approved by the UK Home Office in concordance with UK and European Union law. Ethical approval was granted by the Animal Welfare and Ethical Review Body of the Francis Crick Institute. All experiments conformed to the relevant regulatory standards.

The source of all the transgenic mouse strains used are listed in the Key Resources Table. The *Rac1*^LSL-P29S^ mouse strain described here has been deposited at Jackson Laboratories as JAX stock number 033790 B6.Cg-Rac1<tm1Jdo>/J. Mice were kept and bred in the Biological Research Facility of the Cancer Research UK London Research Institute, which subsequently became the Francis Crick Institute. All strains were kept on a C57BL/6J background. Genotyping was outsourced to an automated genotyping company (Transnetyx). Mice were euthanized when the cumulative tumor diameter reached 15 mm or when mice displayed signs of ill health (weight loss, rapid breathing, hunched posture, piloerection, inactivity).

#### Cell Lines

Cell lines used are listed in the Key Resources Table. Cells were cultured in a humidified incubator at 37°C with a controlled atmosphere of ambient air and 5% or 10% CO_2_. For all cells, growth medium was supplemented with L-glutamine (4 mM), penicillin (100 units/ml) and streptomycin (100 μg/ml). Cells were trypsinized using 0.25% trypsin-EDTA and cryopreserved in growth medium containing 10% dimethylsulfoxide (DMSO; v/v), unless otherwise stated.

The melan-a mouse melanocyte cell line (Bennett et al., 1987) and our own immortalized mouse melanocyte cultures were grown in RPMI with 10% FCS and the mitogen 12-O-tetradecanoylphorbol-13-acetate (TPA) at 200 nM in 5% CO_2_. Three days before freezing and three days after thawing of melanocytes, growth medium was supplemented with 200 μM phenylthiourea (PTU) to inhibit pigmentation, which is thought to hinder recovery from cryopreservation (Godwin et al., 2001). Melanocytes were frozen in growth medium containing 7.5% DMSO.

WM3060 and WM1791C were cultured in MCDB153:Leibovitz L-15 (4:1, v/v), 2% FCS, 1.68 mM CaCl_2_ and 5 μg/ml insulin in 5% CO_2_. YUHEF, YUSOC, YURIF and YUTOGS were cultured in OptiMEM with 5% FCS in 10% CO_2_. The following cell lines were cultured in DMEM with 10% FCS in 10% CO_2_: SK-MEL-2, A2058, C32, A375, IGR-1, 293T, Phoenix-ECO, XB2 and MEFs. The following cell lines were cultured in RPMI with 10% FCS in 5% CO_2_: SK-MEL-5, LOX-IMVI, Colo-792. MCF10A cells were cultured in DMEM:F12 (1:1, v/v) with 5% horse serum, 20 ng/ml EGF, 10 μg/ml insulin and 100 ng/ml cholera toxin in 10% CO_2_. Finally, mouse melanoma cells were cultured in Advanced DMEM/F12 (ThermoFisher) with 5% FCS in 5% CO_2_.

### Methods Details

#### Molecular Cloning

For cloning of pBABE-RAC1 retroviral expression vectors, we used the pcDNA3.1(+)-2xMyc-HRAS^G12V^ construct from the cDNA Resource Center (Missouri; catalogue number RASH00MNC0) as starting point. HRAS^G12V^ was removed using NotI and XbaI restriction sites and replaced with murine RAC1^WT^ cDNA, which was PCR amplified from the pGEX-2T-RAC1 expression vector (Addgene 55692; Fritsch et al., 2013). Site-directed mutagenesis was performed to generate RAC1^P29S^ and RAC1^Q61L^ variants, followed by Sanger sequencing to confirm successful mutagenesis. The RAC1 sequences plus the N-terminal double Myc-tag were PCR amplified and inserted into the retroviral pBABE-hygro vector using BamHI and EcoRI restriction sites.

For generating the pLZRS-ER-RAC1 constructs, we used the pLZRS-ER-HRAS^G12V^ construct (Addgene 21199) as starting point, which was a gifted by Dr. Paul Khavari (Stanford). The ER-HRAS^G12V^ sequence was sub-cloned into the pcDNA3.1(+) expression vector (Invitrogen) using BamHI and NotI restriction sites. Next, the majority of the linker plus HRAS^G12V^ were cut out by restriction digestion using EcoRV and NotI. RAC1^Q61L^ cDNA was PCR amplified from our pcDNA3.1(+)-2xMyc-RAC1^Q61L^ expression construct (see above) and sub-cloned into the digested pcDNA3.1(+)-ER construct using the In-Fusion cloning system (Clontech). This produced a reading frame that starts with the murine ERα domain containing the G525R mutation, followed by a single aspartic acid linker that connects to murine RAC1^Q61^ from the second codon onwards. This construct was used for double site-directed mutagenesis (L61Q and P29S) using the QuickChange Lightning Multi-site Mutagenesis kit (Agilent). After confirming mutagenesis by Sanger sequencing, the ER-RAC1^P29S^ and ER-RAC1^Q61L^ inserts were sub-cloned into pLZRS using BamHI and NotI restriction sites.

PCR amplifications were performed using Primestar HS polymerase (Takara) in combination with oligonucleotides synthesized by Sigma-Aldrich. DNA purification was performed using QIAquick Gel Extraction and PCR Purification kits from Qiagen. Restriction enzymes, CIP alkaline phosphatase and T4 ligase were from NEB. XL1-blue and XL-10 gold competent cells (both from Agilent) were used for transformations and purified DNA preparations were obtained using the Plasmid Miniprep and Maxiprep kits (Qiagen).

#### Isolation and Culture of Primary Mouse Melanocytes

We used the detailed protocol developed by others (Godwin et al., 2001). A variant of the mouse keratinocyte cell line XB2 that can grow without fibroblasts was provided by Dr. Dorothy Bennett (St George’s, University of London). XB2 cells were expanded and treated with mitomycin C at 8 μg/ml to induce growth arrest. Growth-arrested XB2 cells were seeded in 6-well plates in RPMI with 10% FCS in 5% CO_2_ one day before seeding melanocytes to act as ‘feeders’. Neonatal mice aged 2-4 days were euthanized and tail tissue was collected for genotyping. The dorsal skin was sterilized in 70% ethanol, dissected off from the neck to 5 mm above the tail and transferred to ice-cold PBS. Skins were incubated in PBS with 0.5% trypsin and 1 mM EDTA at 37°C for approximately 90-120 minutes. Next, skins were transferred to PBS and the epidermis was split from the dermis using fine forceps and a dissection microscope. The epidermis was washed in PBS, transferred to PBS containing 0.025% trypsin and 500 μM EDTA and finely cut using a scalpel. Trypsin was inactivated using soybean trypsin inhibitor and the minced epidermis was mixed vigorously and seeded on top of the XB2 cells in RPMI with 10% FCS, 200 nM TPA and 200 pM cholera toxin. For the initial ∼3 months, cultures were grown under these conditions and passaged onto fresh XB2 feeders every 1-2 weeks. Medium was replaced twice per week. Cultures were treated with 4OHT at 1 μM from the first to second passage (9-15 days, depending on the growth rate) to specifically recombine the *Rac1*^LSL-P29S^ allele and both *Trp53*^*F*^ alleles in melanocytes and not in contaminating cells. Approximately three months after isolation, proliferation speed increased and cells were cultured without XB2 feeders or cholera toxin, as described in the cell lines section.

#### Isolation of MEFs

Homozygous *Rosa26-CreER* mice were interbred with heterozygous *Rac1*^LSL-P29S^ mice in timed matings. Pregnant females were euthanized between E11.5 and E14.5 to obtain embryos. Heads were used for genotyping. MEFs were isolated and seeded in 6-well plates and cultured as described in the cell lines section. Experiments were performed from the second to fourth passage.

#### Isolation of Mouse Melanoma Cells

Tumor-bearing *Tyr-CreER*^+/-^*;Trp53*^F/F^*;Braf*^CA/WT^*;Rac1*^WT/WT^ and *Tyr-CreER*^+/-^*;Trp53*^F/F^*;Braf*^CA/WT^*;Rac1*^LSL-P29S/WT^ mice were euthanized and tumors were immediately dissected out of the carcass. The epidermis was removed by blunt dissection and deeper, homogenous tumor tissue was isolated, submerged in 70% ethanol for 5 seconds, rinsed in sterile PBS and placed in PBS on ice. The tumor was minced using a scalpel and transferred to 1 to 4 ml digestion solution: HBSS supplemented with 75 ng/ml Liberase TM, 75 ng/ml Liberase TH (both from Roche Applied Science) and 50 U/ml DNAse I (Invitrogen). Tumor tissue was digested at 37°C for 45 minutes to 1 hr in a shaking incubator at 400 rpm. Next, the suspension was diluted in 10 ml growth medium, mixed vigorously, spun down, resuspended and seeded in growth medium.

#### Retroviral Transduction

On the first day, Phoenix-ECO cells were transfected with plasmid DNA using Lipofectamine 2000 (Invitrogen) in antibiotic-free medium. On the second day, medium was refreshed. On the third day, retroviral medium was collected, run through a 0.45 μm filter and either directly used for target cell infection or frozen on dry ice and stored at -80°C. Phoenix-ECO cells received fresh medium to enable a second virus harvest on the fourth day. MCF10A-ECO cells were infected on two consecutive days using retroviral medium:growth medium (1:1 or 2:1, v/v), supplemented with polybrene (Merck Millipore) at 10 μg/ml. The morning following the second infection, medium was replaced with fresh growth medium. Two days after the second infection, cells were trypsinized and re-seeded in a larger vessel in growth medium supplemented with hygromycin B (ThermoFisher) at 200 μg/ml. Selection was maintained for four days, at which time mock-infected control cells were completely eradicated.

#### Lentiviral Transduction

293T cells were co-transfected with pLKO.1-puro shRNA plasmids and the lentiviral packaging plasmids psPAX2 and pMD2.G in antibiotic-free medium using Lipofectamine 2000. The shRNA sequences are listed in the Key Resources Table. The next day, medium was refreshed. On the second day post-transfection, lentiviral medium was collected, run through a 0.45 μm filter, frozen on dry ice and stored at -80°C. Fresh medium was added to the 293T cells and a second lentiviral harvest was performed on the following day. WM3060 target cells were infected with freshly thawed lentiviral medium:growth medium (1:7, v/v), supplemented with polybrene at 3 μg/ml. Two days post-infection, WM3060 cells were trypsinized and re-seeded in a larger vessel in growth medium supplemented with 2 μg/ml puromycin. Cells were selected in puromycin for two days, at which time mock-infected control cells were completely eradicated.

#### siRNA Transfection

siGENOME siRNAs (Dharmacon) were dissolved in siRNA resuspension buffer (Dharmacon) and stored at -80°C. On the day of use, siRNAs were thawed on ice and diluted in HBSS (ThermoFisher) to reach a concentration of 250 nM. Ten μl per well was dispensed onto 96-well plates (Corning or Nunc, ThermoFisher). This solution was mixed with 10 μl HBSS containing 0.1 μl (for mouse melanocytes) or 0.15 μl (for human melanoma cells) DharmaFECT 1 transfection reagent (Dharmacon). The transfection complex was incubated for 20-40 minutes before cells were seeded on top in a volume of 80 μl.

#### Chemical Inhibitors in Human Melanoma Cell Lines

Drugs and the suppliers from which they were obtained are listed in the Key Resources Table. For the drug treatments, (5Z)-7-Oxozeaenol, Avagacestat, NSC-23766, CK-869 and CK-666 were applied in an earlier version of our melanoma cell line panel. This panel contained five RAC1^WT^ cell lines and five RAC1^P29S^ cell lines. Specifically, cell lines A375, SK-MEL-5, YUHEF and YURIF were not used, as opposed to the full melanoma cell line panel (see Figure S4I). Genotypes for the main melanoma driver genes were obtained from publicly available sequencing data from the COSMIC database (Wellcome Sanger Institute), the CCLE database (Broad) and publications (Garman et al., 2017, Krauthammer et al., 2015).

#### Viability Assays

For the crystal violet assay, cells were washed once with PBS before fixing and staining by submerging the monolayer in crystal violet solution (0.2% crystal violet, 2% ethanol in H_2_O) and gently agitating plates for approximately 30 minutes. The monolayer was washed five times using H_2_O and dried overnight. Wells were photographed on a flatbed scanner. For quantification, crystal violet was dissolved in 10% acetic acid and absorbance was measured at 595 nm using a plate reader.

For the CellTiter-Blue assay (Promega), cells were seeded in clear 96-well Nunc plates (ThermoFisher) and cultured in 100 μl medium. At the end of the experiment, 5 μl CellTiter-Blue reagent was added to each well and plates were returned to their incubator for 90 minutes. Fluorescence was measured using an EnVision plate reader (PerkinElmer) with an excitation wavelength of 560 nm and an emission wavelength of 590 nm. The average value of wells containing only medium was subtracted from values of wells containing cells.

For the CellTiter-Glo assay (Promega), cells were seeded in black, clear-bottom 96-well plates (Corning) and cultured in 100 μl medium. At the end of the experiment, 50 μl freshly dissolved CellTiter-Glo lysis buffer was added to each well using a multichannel pipet and plates were incubated at room temperature on a plate-shaker for twenty minutes. Bioluminescence was measured using an EnVision plate reader (PerkinElmer).

#### Apoptosis Assay

Cells were seeded in clear 96-well Nunc plates (ThermoFisher) and cultured in 100 μl medium. Apoptosis induction was determined using a caspase 3/7 consensus site peptide (Z-DEVD) conjugated to rhodamine 110 (Invitrogen). 100 μl of apoptosis solution (containing 9 μg/ml peptide, 5 mM DTT, 0.1% CHAPS, 25 mM Hepes pH 7.3, 1 mM EDTA and 100 mM NaCl) was added to each well and incubated at room temperature for 5 hr. Fluorescence was measured using an EnVision plate reader (PerkinElmer) with an excitation wavelength of 485 nm and an emission wavelength of 535 nm. Prior to the determination of apoptosis, relative cell viability of each well was measured using CellTiter-Blue assay (as described above).

#### F-actin Staining Assay

Cells were seeded in 8-well tissue culture-treated glass chamber slides (Falcon). Prior to fixation, monolayers were briefly rinsed twice with pre-warmed PBS and fixed with 3.8% paraformaldehyde (ThermoFisher) in PBS for 10 minutes. Cells were permeabilized with 0.1% Triton X-100 (Sigma-Aldrich) in PBS for 4 minutes, washed twice with PBS and pre-incubated with 1% BSA (Sigma-Aldrich) in PBS for 30 minutes. F-actin was visualized with a Leica SP5 inverted confocal microscope following incubation of slides with approx. 3 nM Alexa Fluor 488 phalloidin (ThermoFisher) in 1% BSA in PBS for 30 minutes, two washes in PBS and mounting with ProLong Antifade (ThermoFisher). Representative z stack images were taken under oil immersion with a 63x objective.

#### Sphere Formation Assay

Single cell suspensions were seeded in growth medium containing 0.3% agar on top of a layer of 0.6% agar in 6-well plates. One ml growth medium was put on top and refreshed every three days. Three weeks after cells were seeded, the growth medium on top of the agar layers was replaced with 0.1% Giemsa dye in glycerol:methanol (5:24, v/v). Colonies were stained under gentle agitation for 20 minutes. Next, agar was destained by washing five times with H_2_O for approximately 30 minutes, followed by a sixth wash overnight. Plates were scanned using a flatbed scanner at 1200 dpi. Grayscale images were opened in ImageJ, a size and intensity threshold was set to distinguish colonies from background and colonies were counted automatically using the analyze particles function.

#### *Rac1*^*LSL-P29S*^ Recombination Assay

Genomic DNA was isolated using the DNeasy Blood & Tissue Kit (Qiagen) and the DNA concentration in the elute was estimated using a NanoDrop analyzer (ThermoFisher). Fifty ng DNA was used in a 50 μl PCR reaction using the PfuTurbo DNA polymerase (Agilent) in combination with Rac1-FP5 and Rac1-RP5 primers (sequences listed in the Key Resources Table), which flank the LSL cassette. We used the following PCR program: 98°C for 1’ → 98°C for 30", 66°C for 30", 72°C for 90" (35 cycles) → 72°C for 10'. The product was run on a 1.2% agarose gel to separate the 453 bp band corresponding to *Rac1*^WT^ from the recombined *Rac1*^Lox-P29S^ band, which is 34 bp larger as a consequence of the single LoxP motif.

#### Quantitative PCR

Total RNA was isolated using the RNeasy kit (Qiagen) and eluted in 30 μl H_2_O. RNA concentrations were estimated using a NanoDrop analyzer (ThermoFisher). One μg purified RNA was reverse transcribed using the Maxima first strand cDNA synthesis kit (ThermoFisher) and diluted to a volume of one ml H_2_O. Four μl of this solution was used in a ten μl qPCR reaction using the Fast SYBR Green FAST Master Mix (Applied Biosystems) in combination with QuantiTect primer pairs (Qiagen). Reactions were carried out in 384-well plates using the QuantStudio 7 system (Applied Biosystems). The ΔΔCt method was used to calculate relative expression values, which were normalized using the expression of housekeeping genes *Gapdh* and *Tbp*.

#### Immunoblotting

Cells were seeded in 6-well plates. At the end of the experiment, plates were placed on ice, medium was aspirated and the monolayer washed twice with ice-cold PBS before adding 100-150 μl lysis buffer (1% Triton X-100, 50 mM HEPES at pH 7.4, 0.15 M NaCl, 1.5 mM MgCl_2_, 1 mM EGTA, 0.1M NaF, 10 mM Na pyrophosphate, 1 mM Na_3_VO_4_, 10% glycerol, PhosSTOP phosphatase inhibitor cocktail and EDTA-free cOmplete protease inhibitor cocktail, both added fresh to the lysis buffer and from Roche Applied Science). Monolayers were incubated on ice in lysis buffer for 10-20 minutes and tilted every two minutes, before scraping with a rubber policeman and collection of the lysate in pre-cooled microcentrifuge tubes on ice. For tumors, tissue was isolated directly after sacrificing animals. The epidermis was dissected off to ensure pure tumor tissue, which was snap-frozen in liquid N_2_ and stored at -80°C. Frozen tumor tissue was then submerged in lysis buffer and immediately homogenized with a microfuge pestle on ice. Lysates were vortexed for five seconds and spun in a table-top centrifuge at 4°C for ten minutes at 21,000 g. The supernatant was transferred to a new microcentrifuge tube on ice and the pellet was discarded. Protein concentrations were estimated using the Bradford assay (Bio-Rad) with bovine serum albumin as a standard. NuPAGE LDS lysis buffer (ThermoFisher) was added and lysates briefly vortexed before incubation at 72°C for eight minutes. Lysates were either directly used for SDS-PAGE or stored at -80°C and re-heated directly before use. Equal amounts of protein (generally 15-25 μg) were loaded onto NuPAGE 4-12% Bis-Tris protein gels (ThermoFisher) and SDS-PAGE was performed using Bio-Rad equipment according to the Cell Signaling Technologies protocol (available on their website). Immunoblots were developed using either the Odyssey Imaging System (LI-COR) or using secondary antibodies conjugated to horseradish peroxidase (GE Healthcare), before chemiluminescent visualization using standard film or the ImageQuant LAS 4000 or the Amersham Imager 600 (both from GE Healthcare). Primary antibodies are listed in the Key Resources Table.

#### PAK1 Binding Domain Pull-Down Assay

The RAC1 activity assay kit from Merck Millipore was used. Cells were seeded in 10 cm dishes (Corning). Growth medium was replaced with growth factor-reduced medium 24 hr before lysis. For MCF10A cells, this is 1% horse serum without addition of EGF or insulin. For melan-a, this is growth medium without addition of TPA. At the end of the experiment, monolayers were washed twice with ice-cold PBS, placed on ice and lysed in MLB supplemented with phosphatase and protease inhibitor cocktails (Roche Applied Science). Monolayers were scraped off using a rubber policeman and collected in a pre-cooled microcentrifuge tube on ice. The PAK1 binding domain pull-down assay was performed according to the manufacturer’s protocol and immunoblotting was performed as described above.

#### Reverse-Phase Protein Array

Cells were seeded in 6-well plates in full medium. Twenty-four hr before lysis, full medium was aspirated, the monolayer washed with PBS and replaced with medium lacking TPA. To activate RAC1, ER-RAC1^P29S^ melanocytes were treated with 4OHT at 500 nM for 40 hr. Protein lysates (100 μl) were collected and protein concentrations estimated using the Bradford assay. Lysis buffer was added to reach a final concentration of 1.33 μg/ml. Next, 4X SDS sample buffer was added (40% Glycerol, 8% SDS, 250 mM Tris-HCL at pH 6.8, plus 10% 2-mercaptoethanol added fresh). Lysates were vortexed, incubated for 5 minutes at 95°C and stored at -80°C. The experiment was performed three times on a different day to obtain three independent replicates. Lysates were shipped on dry ice to the RPPA Core Facility at the MD Anderson Cancer Center, where the reverse-phase protein array (RPPA) was performed according to their standardized protocol. In brief, lysates were serially diluted, arrayed on slides with a 2470 Microarray Printer (Aushon Biosystems) and probed with 300 validated (phospho-)antibodies and biotinylated secondary antibodies. Antibody binding was measured colorimetrically using tyramide dye. Data were analyzed using Array-Pro Analyzer software (MediaCybernetics) and the SuperCurve GUI (MD Anderson). To correct for total protein loading, values for individual antibodies were normalized using values from the entire antibody panel.

#### RNA-seq of Melanocytes with ER-RAC1^P29S^

Cells were cultured in melanocyte growth medium. Twenty-four hr before lysis, monolayers were washed once with PBS and medium was changed to medium lacking TPA. Cells were lysed and total RNA was isolated using the RNeasy Mini Kit (Qiagen). The experiment was performed three times on a different day to obtain three independent replicates. RNA integrity was verified using the RNA 6000 Nano Kit in combination with the 2100 Bioanalyzer (both from Agilent). Next, mRNA libraries were prepared with the KAPA mRNA Hyper Prep kit (Roche Applied Science) and sequenced as 9-plex pools on the HiSeq 4000 (Illumina). Data were analyzed as described in the quantification and statistical analysis section.

#### RNA-seq of Melanocytes with Endogenous RAC1^P29S^

Mouse melanocytes were isolated and cultured as described in above. Three independent melanocyte cultures from *Tyr-CreER*^+/-^*;Trp53*^F/F^*;Rac1*^LSL-P29S/WT^ mice were used and compared to two independent cultures from *Tyr-CreER*^+/-^*;Trp53*^F/F^*;Rac1*^WT/WT^ mice. Complete recombination of the *Rac1*^LSL-P29S^ and *Trp53*^F^ alleles was confirmed beforehand. The spontaneously immortalized C57BL/6J melanocyte cell line melan-a (Bennett et al., 1987) was used as the third independent replicate for the *Rac1*^WT/WT^ group. 24 hr prior to lysis, cell monolayers were washed with PBS and medium was changed to growth medium lacking TPA. RNA was isolated and sequenced as described above and data were analyzed as described in the quantification and statistical analysis section.

#### RNA-seq of Tumor Lysates

Animals were euthanized and tumors harvested. The epidermis was dissected off and a piece of central homogenous tumor tissue weighing approximately 50 mg was isolated and snap-frozen in liquid N_2_. Tissue was stored at -80°C prior to lysis by submersion in RLT buffer (Qiagen), directly followed by homogenization in a microcentrifuge tube using a pestle. RNA was isolated and sequenced as described above and data were analyzed as described in the quantification and statistical analysis section.

#### Fluorescence Flow Cytometric Analysis

Animals were euthanized and the spleen and mesenteric lymph nodes were collected in ice-cold PBS. Tissue was crushed on top of a 70 μm cell strainer with a plunger from a 5 ml syringe and washed through using ice-cold PBS. Cells were spun down at 1500 rpm for 5 minutes at 4°C. The supernatant was discarded and the pellet resuspended in FCS:DMSO (9:1, v/v). Cells were stored in cryovials in liquid N_2_ until the day of analysis. Before analysis, cells were thawed, washed in ice-cold FACS buffer (PBS with 2% FCS), resuspended in FACS buffer on ice and counted using a Vi-CELL Counter (Beckman Coulter). Maximum 10^6^ cells were allocated per well in 200 μl FACS buffer. Cells were washed once, spun down at 2000 rpm for two minutes and stained with 50 μl antibody cocktail. Antibodies were diluted 1:200 and are listed in the Key Resources Table. Zombie NIR Live/Dead Dye (Biolegend) was used to stain for live cells. Cells were stained for 30 minutes at 4°C and washed twice in FACS Buffer at 2000 rpm for two minutes at 4°C. Samples were kept on ice until acquisition on a BD LSRFortessa analyzer (BD Biosciences). Data were analyzed using FlowJo 10.

#### RAC1 Co-dependencies in Human Cancer Cells

Complete gene-level dependency score datasets were obtained from the Project Achilles webportal (Tsherniak et al., 2017) and directly from Dr. Tobias Schmelzle (Novartis; ATARiS values; McDonald et al., 2017). We identified genes correlating with RAC1 dependency scores by Spearman’s correlation test. Resulting *p* values were corrected for multiple testing using Benjamini-Hochberg to obtain an FDR value (threshold 0.05).

#### Mutational Landscape of RAC1^P29S/L^ Human Melanoma

Whole exome sequencing data of 537 patient melanomas from four large studies (Hodis et al., 2012, Hugo et al., 2016, Krauthammer et al., 2012, The Cancer Genome Atlas Network et al., 2015) was extracted from cBioPortal. In addition, sequencing data from 607 melanoma cases was obtained from AACR Project GENIE v3.0.0 (AACR Project GENIE Consortium, 2017), also via cBioPortal. Finally, whole exome sequencing data from eight more *RAC1*^P29S^ cases was mined directly from publications (Krauthammer et al., 2015, Wagle et al., 2014) and one *RAC1*^P29S^ case from the CCLE database (Broad). For the mutation rate analysis of the total cutaneous melanoma cohort, only the complete datasets from cBioPortal could be used (n = 1144). Tendency towards co-occurrence of two mutations within the same tumor was tested via cBioPortal using log odds ratio testing in combination with Fisher’s exact test. For this purpose, the complete cutaneous melanoma collection on cBioPortal (n = 709) and the AACR Project GENIE v3.0.0 database (n = 1868) were applied independently.

#### Publicly-Available Survival Data

Survival data of the TCGA melanoma cohort were obtained from cBioPortal. Relapse-free survival refers to the time from the initial melanoma diagnosis to relapse. Melanoma tissue for sequencing was not necessarily procured at diagnosis.

#### Mouse Embryo Analysis

*PGK-Cre*^+/-^ females were set up with *Rac1*^LSL-P29S/WT^ males in timed matings. Females were euthanized and embryos quickly dissected out of the uterus. The yolk sac was used for genotyping. At E8.5, embryos were considered viable if there were no gross morphological or size abnormalities. From E9.5 onwards, embryos were considered viable if a heartbeat was observed. Embryos were fixed in neutral buffered formalin (10%) for 24 hr and transferred to 70% ethanol for photography.

#### Systemic Tamoxifen Treatment

Tamoxifen was dissolved in ethanol at 55°C at 300 mg/ml, aliquoted and stored at -80°C. At the day of use, aliquots were mixed with 9 parts (v/v) sunflower oil and gently shaken at 55°C until a homogenous solution was obtained. Adult mice were treated with 150 mg/kg tamoxifen by oral gavage on three instances over the course of five days, with a rest day in between treatments. Both groups contained similar numbers of male and female mice.

#### Topical 4OHT Treatment

A patch on the central-lower dorsal skin of approximately 3 x 2 cm was shaven the day before treatment. On the day of treatment, 4OHT was freshly dissolved in DMSO at 50 mg/ml. Fifteen μl 4OHT solution was applied to the shaven skin with a pipette and dispersed using a paintbrush. In this way, animals were treated for three times over the course of seven days, with at least one rest day in between treatments. Adult male and female mice were used and similarly represented across groups.

#### Histology

Tissues were fixed in 10% neutral buffered formalin shortly after euthanasia. After 24 to 48 hr, fixed tissues were transferred to 70% ethanol. Next, tissues were embedded in paraffin and 4 μm sections were mounted on slides. Hematoxylin and eosin (H&E) staining was performed automatically using a Tissue-Tek Prisma 6132 (Sakura). For S100, SOX10 and B220 immunohistochemistry, antigen retrieval was performed by microwaving in citrate buffer (pH 6.0). Slides were incubated with primary antibodies for 1 hr at room temperature in 1:75, 1:50 and 1:200 dilutions, respectively. For p16 and p27, antigen retrieval was performed by microwaving in a 50 mM Tris, 2 mM EDTA buffer (pH 9.0). Slides were incubated with primary antibodies overnight at a 1:250 dilution for p16 and a 1:50 dilution for p27. Primary antibodies used are listed in the Key Resources Table.

*In situ* hybridization was performed using the RNAscope assay according to manufacturer’s instructions (ACDBio). Tissues were fixed, embedded in paraffin, sectioned and then stained using the RNAscope 2.5 LS Reagent Kit (red) in combination with the BOND RX automated staining system (Leica Biosystems). For all samples, probes targeting *Actr3* and *Cyr61* were applied in combination with *PPIB* as a positive control and *DapB* as a negative control.

#### Mouse Drug Treatment

PLX4720 was a gift from Plexxicon. The drug was incorporated into food pellets at 200 ppm (Research Diets, Inc.), resembling clinical exposure in melanoma patients treated with vemurafenib. The same diet was prepared without PLX4720 for control purposes. *Tyr-CreER*^+/-^*;Pten*^F/WT^*;Braf*^CA/WT^*;Rac1*^WT/WT^ were treated with 4OHT ∼7 weeks before *Tyr-CreER*^+/-^*;Pten*^F/WT^*;Braf*^CA/WT^*;Rac1*^LSL-P29S/WT^ mice to ensure both groups developed tumors and were ready for treatment at the same time. When the majority of mice had at least one tumor of several mm, regular chow was replaced with either the PLX4720 diet or the control diet. Tumors were measured every five days using a caliper. Only tumors >2 mm were considered. CCG-257081 SRF/MRTF inhibitor was given to mice by oral gavage at a dose of 100 mg/kg q.d. The drug was dissolved in a vehicle of 30% PEG, 5% DMSO in phosphate buffered Saline.

### Quantification and Statistical Analysis

#### Statistical Analysis

For statistical comparison of two groups, F test was performed to test for equality of variance. If variance of groups was significantly different, the Mann-Whitney test was used. If not, an unpaired two-tailed t-test was used. To correct for a small number of repeated tests, Holm-Sidak correction was used. To correct for a large number of multiple tests, we applied Benjamini and Hochberg correction with the FDR Q value at 5%. To compare two growth curves, two-way ANOVA was performed. To compare read counts of individual genes in mRNA-seq datasets of two groups, Wald test was used with a Benjamini and Hochberg correction with an FDR Q value of 5%. Chi-square test was used to statistically assess differences between expected and observed genotypes. To compare two survival curves, the Mantel-Cox log-rank test was used. Statistical analyses were performed in Prism 7 (GraphPad Software) or in R.

Unless otherwise stated, on bar graphs and line graphs, values represent means ± SD for n = 3 biological replicates and are representative of at least three independently repeated experiments.

#### RNA Sequencing Analysis

Raw reads were quality and adapter trimmed using cutadapt-1.9.1 prior to alignment. Reads were then aligned and quantified using RSEM-1.2.31/STAR-2.5.2 (Dobin et al., 2013, Li and Dewey, 2011) against the mouse genome GRCm38 and annotation release 86, both from Ensembl. Differential gene expression analysis was performed in R using the DESeq2 package (Love et al., 2014). Differential genes were selected using a 0.05 false-discovery rate (FDR) threshold. For gene set enrichment analysis (GSEA), we used the Java desktop application in combination with pre-ranked gene expression lists that were ordered using the Wald statistic. To test for enrichment of human gene sets from MSigDB (Broad) in our mouse gene expression data, mouse gene identifiers were translated to homologous human gene identifiers using the HomoloGene database (NCBI). If there was no human homolog in the database, the gene was removed from the analysis. SRF/MRTF/TCF target gene sets from Esnault et al. (2014) and Gualdrini et al. (2016) were murine, hence translation was not required. Due to the great sequencing depth and cellular heterogeneity of the tumor microenvironment, reads from almost the entire exome were detected in tumor lysates (n = 19750 genes). Many genes were expressed at very low levels in a single or minority of samples. To focus on biologically relevant genes, our analyses of tumor lysates only included genes with a mean normalized TPM value ≥ 1 in both *Rac1*^*WT/WT*^ and *Rac1*^*LSL-P29S/WT*^ groups (n = 14460 genes included in the analysis). Gene expression signatures for senescence (Fridman and Tainsky, 2008), ageing (de Magalhaes et al., 2009) and E2F targets were obtained from MSigDB (Broad). The SASP signature was obtained from a publication (Coppe et al., 2010).

### Data and Software Availability

Messenger RNA sequencing data was deposited to the NCBI Gene Expression Omnibus (GEO) repository (identifiers GEO: GSE118349, GSE118343, GSE118344). R scripts used to analyse data are available at https://github.com/juliandownward.

## Supplemental Information

Document S1. Figures S1–S7

Document S2. Article plus Supplemental Information
