# Supplementary material for: RAC1P29S Induces a Mesenchymal Phenotypic Switch via Serum Response Factor to Promote Melanoma Development and Therapy Resistance
Source: Cancer Cell. 2019 Jul 8;36(1):68–83.e9. doi: 10.1016/j.ccell.2019.05.015 (PMC6617390; doi:10.1016/j.ccell.2019.05.015)
Supplement: Document S1. Figures S1–S7 [file mmc1.pdf]

Cancer Cell, Volume *36*

Supplemental Information

RAC1^{P29S} Induces a Mesenchymal Phenotypic Switch via Serum Response Factor to Promote Melanoma Development and Therapy Resistance

Daniël A. Lionarons, David C. Hancock, Sareena Rana, Philip East, Christopher Moore, Miguel M. Murillo, Joana Carvalho, Bradley Spencer-Dene, Eleanor Herbert, Gordon Stamp, Djamil Damry, Dinis P. Calado, Ian Rosewell, Ralph Fritsch, Richard R. Neubig, Miriam Molina-Arcas, and Julian Downward

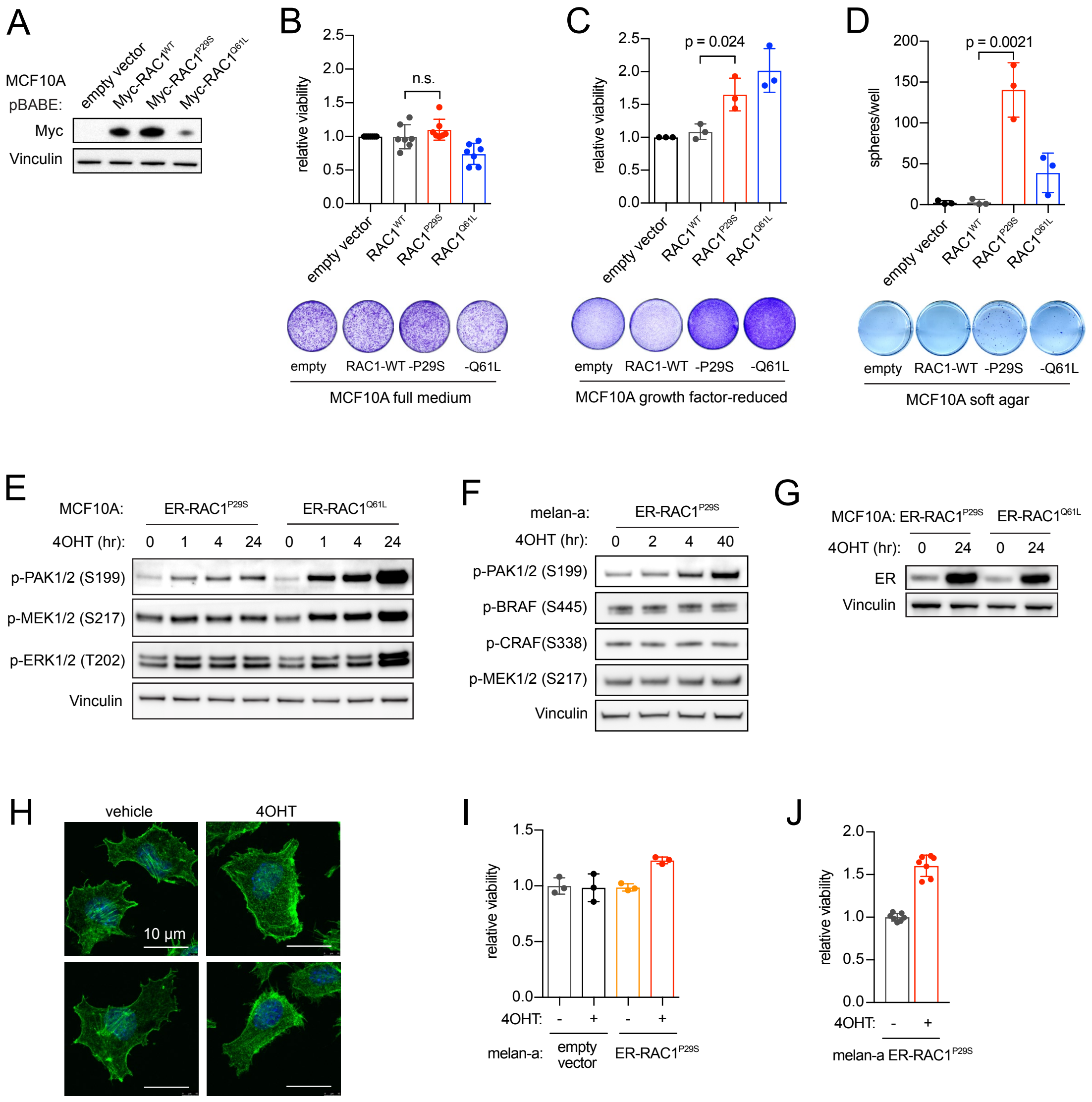

Figure S1. Effect of activation of RAC1^{P29S} on survival of epithelial cells and melanocytes.

Related to Figure 1.

(A) Stable expression of Myc epitope tagged-RAC1 in MCF10A cells following retroviral transduction.

(B) The effect of RAC1^{P29S} on MCF10A normal immortalized breast epithelial cell proliferation in full medium. Cells were seeded at equal densities and stained with crystal violet after 5 days. Representative wells are shown. Crystal violet was dissolved and absorbance measured (n = 7 independent experiments; n.s., not statistically significant for t-test).

(C) The effect of RAC1^{P29S} on MCF10A cell proliferation in growth factor-reduced medium, which contained 1% horse serum without addition of EGF or insulin. Cells were seeded at equal densities and stained with crystal violet after 1 week.

Representative wells are shown. Crystal violet was dissolved and absorbance measured (n = 3 independent experiments, p value of t-test shown).
(D) Sphere formation in soft agar of MCF10A cells with $RAC1^{P29S}$. Equal densities of cells were seeded in soft agar and grown for 3 weeks. Spheres were stained and counted automatically (n = 3 independent experiments, p value of t-test shown).
(E) Signaling in MCF10A cells with ER-$RAC1^{P29S}$ and ER-$RAC1^{Q61L}$. Twenty-four hr before lysis, cells were placed in growth factor-reduced medium, which contained 1% horse serum without addition of EGF or insulin.
(F) MAPK signaling in melan-a melanocytes with ER-$RAC1^{P29S}$, as determined by immunoblotting. Phospho-PAK1/2 is included to demonstrate activation of ER-$RAC1^{P29S}$.
(G) Direct comparison of protein expression of activated ER-$RAC1^{P29S}$ with activated ER-$RAC1^{Q61L}$. MCF10A cells were cultured in growth factor-reduced medium and treated with 4OHT at 500 nM before immunoblotting with the indicated antibodies.
(H) Fluorescence confocal microscopy using phalloidin to stain for polymerized actin in melan-a melanocytes with ER-$RAC1^{P29S}$ treated with 500 nM 4OHT for 48 hr.
(I) Proliferation of melan-a mouse melanocytes with active ER-$RAC1^{P29S}$ grown in full medium for 3 days. Viability was quantified using CellTiter-Blue (n = 3 biological replicates).
(J) Proliferation of melan-a mouse melanocytes with activated ER-$RAC1^{P29S}$ cultured in ultra-low adhesion plates in full medium, with or without 4OHT at 500 nM. After 3-5 days, viability was quantified using CellTiter-Glo (n = 7 biological replicates from 2 independent experiments).
Wherever used, error bars represent means ± SD.

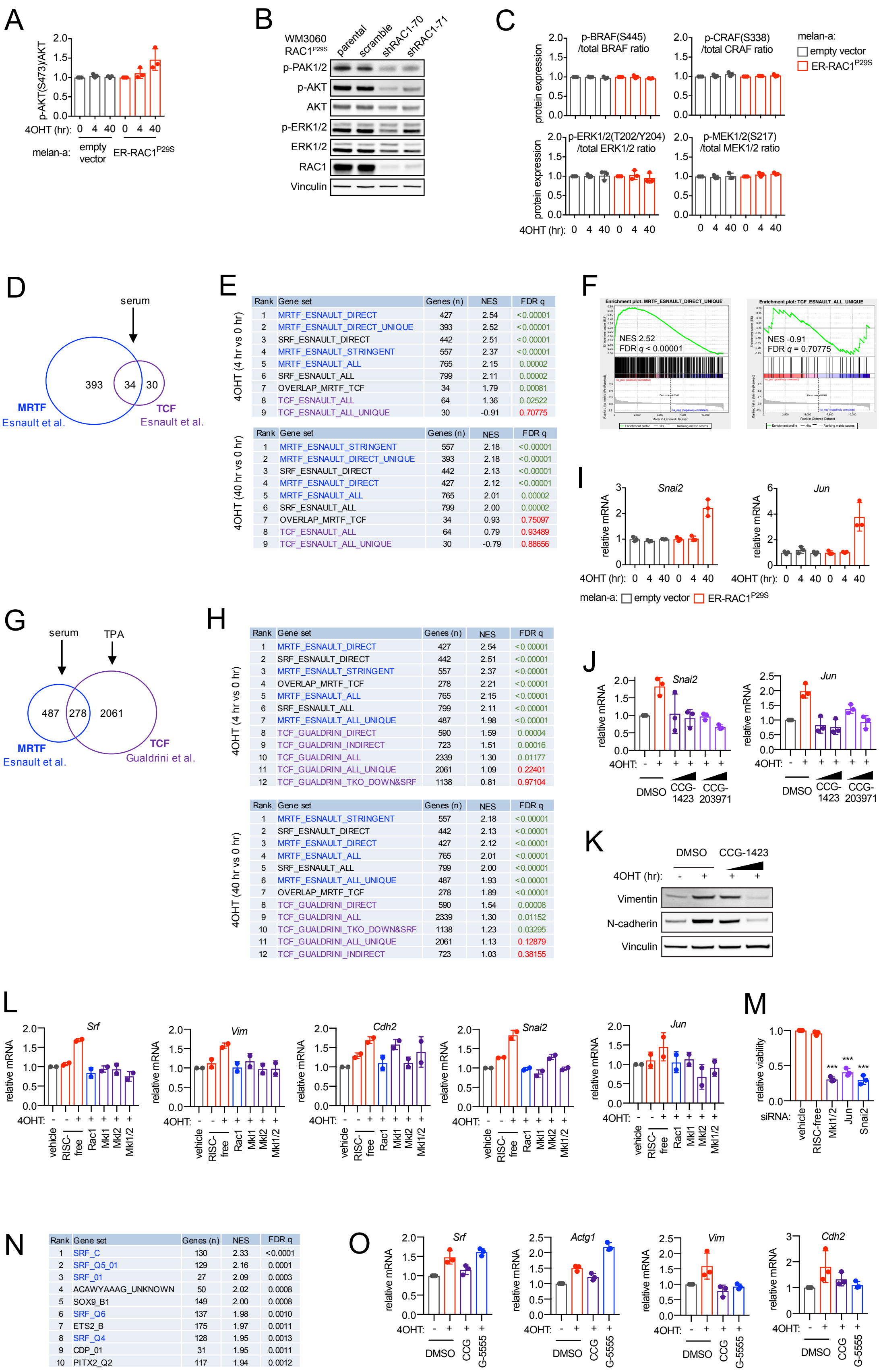

E — 4OHT (4 hr vs 0 hr)

Rank	Gene set	Genes (n)	NES	FDR q
1	MRTF_ESNAULT_DIRECT	427	2.54	<0.00001
2	MRTF_ESNAULT_DIRECT_UNIQUE	393	2.52	<0.00001
3	SRF_ESNAULT_DIRECT	442	2.51	<0.00001
4	MRTF_ESNAULT_STRINGENT	557	2.37	<0.00001
5	MRTF_ESNAULT_ALL	765	2.15	0.00002
6	SRF_ESNAULT_ALL	799	2.11	0.00002
7	OVERLAP_MRTF_TCF	34	1.79	0.00081
8	TCF_ESNAULT_ALL	64	1.36	0.02522
9	TCF_ESNAULT_ALL_UNIQUE	30	-0.91	0.70775

E — 4OHT (40 hr vs 0 hr)

Rank	Gene set	Genes (n)	NES	FDR q
1	MRTF_ESNAULT_STRINGENT	557	2.18	<0.00001
2	MRTF_ESNAULT_DIRECT_UNIQUE	393	2.18	<0.00001
3	SRF_ESNAULT_DIRECT	442	2.13	<0.00001
4	MRTF_ESNAULT_DIRECT	427	2.12	<0.00001
5	MRTF_ESNAULT_ALL	765	2.01	0.00002
6	SRF_ESNAULT_ALL	799	2.00	0.00002
7	OVERLAP_MRTF_TCF	34	0.93	0.75097
8	TCF_ESNAULT_ALL	64	0.79	0.93489
9	TCF_ESNAULT_ALL_UNIQUE	30	-0.79	0.88656

H — 4OHT (4 hr vs 0 hr)

Rank	Gene set	Genes (n)	NES	FDR q
1	MRTF_ESNAULT_DIRECT	427	2.54	<0.00001
2	SRF_ESNAULT_DIRECT	442	2.51	<0.00001
3	MRTF_ESNAULT_STRINGENT	557	2.37	<0.00001
4	OVERLAP_MRTF_TCF	278	2.21	<0.00001
5	MRTF_ESNAULT_ALL	765	2.15	<0.00001
6	SRF_ESNAULT_ALL	799	2.11	<0.00001
7	MRTF_ESNAULT_ALL_UNIQUE	487	1.98	<0.00001
8	TCF_GUALDRINI_DIRECT	590	1.59	0.00004
9	TCF_GUALDRINI_INDIRECT	723	1.51	0.00016
10	TCF_GUALDRINI_ALL	2339	1.30	0.01177
11	TCF_GUALDRINI_ALL_UNIQUE	2061	1.09	0.22401
12	TCF_GUALDRINI_TKO_DOWN&SRF	1138	0.81	0.97104

H — 4OHT (40 hr vs 0 hr)

Rank	Gene set	Genes (n)	NES	FDR q
1	MRTF_ESNAULT_STRINGENT	557	2.18	<0.00001
2	SRF_ESNAULT_DIRECT	442	2.13	<0.00001
3	MRTF_ESNAULT_DIRECT	427	2.12	<0.00001
4	MRTF_ESNAULT_ALL	765	2.01	<0.00001
5	SRF_ESNAULT_ALL	799	2.00	<0.00001
6	MRTF_ESNAULT_ALL_UNIQUE	487	1.93	<0.00001
7	OVERLAP_MRTF_TCF	278	1.89	<0.00001
8	TCF_GUALDRINI_DIRECT	590	1.54	0.00008
9	TCF_GUALDRINI_ALL	2339	1.30	0.01152
10	TCF_GUALDRINI_TKO_DOWN&SRF	1138	1.23	0.03295
11	TCF_GUALDRINI_ALL_UNIQUE	2061	1.13	0.12879
12	TCF_GUALDRINI_INDIRECT	723	1.03	0.38155

N

Rank	Gene set	Genes (n)	NES	FDR q
1	SRF_C	130	2.33	<0.0001
2	SRF_Q5_01	129	2.16	0.0001
3	SRF_01	27	2.09	0.0003
4	ACAWYAAAG_UNKNOWN	50	2.02	0.0008
5	SOX9_B1	149	2.00	0.0008
6	SRF_Q6	137	1.98	0.0010
7	ETS2_B	175	1.97	0.0011
8	SRF_Q4	128	1.95	0.0013
9	CDP_01	31	1.95	0.0011
10	PITX2_Q2	117	1.94	0.0012

Figure S2. Effect of acute activation of RAC1^{P29S} in melanocytes on induction of AKT and SRF/MRTF effector pathways.

Related to Figure 2.

(A) Phospho-AKT (S473) levels normalized using pan-AKT as determined by the reverse-phase protein array. Bars represent means ± SD (n = 3 independent experiments).

(B) Role of RAC1 in activation of the AKT pathway in WM3060, a human melanoma cell line with endogenous RAC1^{P29S}. RAC1 was depleted with shRNAs integrated into the genome using the lentiviral pLKO vector. Seven days post-infection, cells were lysed for immunoblotting.

(C) Effect of activation of ER-RAC1^{P29S} on the MAPK pathway in melanocytes. Reverse-phase protein array data shown, bars represent means ± SD (n = 3 independent experiments).

(D) Schematic of overlap between MRTF and TCF target gene sets induced by serum (Esnault et al., 2014).

(E) Gene set enrichment analysis (GSEA) analysis using MRTF and TCF target gene sets in combination with RNA-seq data from ER-RAC1^{P29S} melan-a melanocytes. The OVERLAP_MRTF_TCF gene set represents the overlap between MRTF_ESNAULT_DIRECT and TCF_ESNAULT_ALL gene sets. It was not possible to test overlap using MRTF_ESNAULT_ALL gene set because that leaves just 8 unique genes in TCF_ESNAULT_ALL.

(F) GSEA plots for selected gene sets from the GSEA analysis presented in panel H.

(G) Schematic of overlap between MRTF target genes induced by serum (Esnault et al., 2014) and TCF target genes induced by TPA treatment (Gualdrini et al., 2016).

(H) GSEA analysis using MRTF and TCF target gene sets in combination with RNA-seq data from melan-a ER-RAC1^{P29S} melanocytes. The OVERLAP_MRTF_TCF gene set represents the overlap between MRTF_ESNAULT_ALL and TCF_GUALDRINI_ALL gene sets.

(I) Effect of activation of ER-RAC1^{P29S} on the SRF/MRTF targets *Snai2* and *Jun*, which encode transcription factors involved in EMT. Normalized RNA-seq reads shown. Bars represent means ± SD of 3 independent experiments.

(J) Role of SRF/MRTF in induction of *Snai2* and *Jun* gene expression by ER-RAC1^{P29S}. Cells were treated with 5-10 μM CCG-1423 or CCG-203971 one hour before 4OHT (1 μM), which was maintained for 48 hr. Twenty-four hr before lysis, cells were starved of TPA. mRNA levels were determined using qPCR and normalized to *Gapdh* expression. Bars represent means ± SD (n = 3 independent experiments).

(K) Effect of treatment with SRF/MRTF pathway inhibitors on mesenchymal marker induction by ER-RAC1^{P29S}. Cells were pre-treated with 5 or 10 μM CCG-1423 one hour before 4OHT treatment (1 μM), which was maintained for 48 hr. Twenty-four hr before lysis, cells were starved of TPA. Immunoblotting was performed using the indicated antibodies.

(L) Effect of genetic depletion of MRTF-A/B (Mkl1/2) on gene expression of EMT transcription factors and mesenchymal marker in melanocytes with activated ER-RAC1^{P29S}. Cells were cultured in full medium and treated with 1 μM 4OHT and transfected with the indicated siRNAs for 48 hr. Twenty-four hr before lysis, cells were starved of TPA. mRNA levels were determined using qPCR; bars represent means ± SD (n = 2 independent experiments).
(M) Effect of depletion of transcription factors involved in EMT on survival ability of melanocytes with activated RAC1^{P29S}. Viability of melanocytes with ER-RAC1^{P29S} in growth factor-reduced medium, which contained 0.25% FCS without TPA. Cells were seeded treated with 1 μM 4OHT and transfected with the indicated siRNAs. Seventy-two hr post-transfection, viability was quantified using CellTiter-Blue. Bars represent means ± SD (n = 3 independent experiments); t-test versus RISC-free was used with Holm-Sidak correction for multiple testing; ***, $p < 0.001$.
(N) Transcription factor target enrichment analysis of gene expression changes in melanocytes 40 hr after activation of ER-RAC1^{P29S}. GSEA was performed using the transcription factor target gene sets from the MSigDB database (Broad) on gene expression changes in ER-RAC1^{P29S} melanocytes treated for 40 hr with 4OHT compared to 0 hr control. NES, normalized enrichment score; genes (n), number of genes in the gene set also present in the expression data.
(O) Effect of inhibition of PAK on gene expression of SRF/MRTF targets and mesenchymal markers downstream of ER-RAC1^{P29S}. Cells were cultured in full medium and treated with 5 μM CCG-203971 or 1 μM G-5555 one hour before 4OHT treatment (1 μM), which was maintained for 48 hr. Twenty-four hr before lysis, cells were starved of TPA. mRNA levels were determined using qPCR; bars represent means ± SD (n = 3 independent experiments).

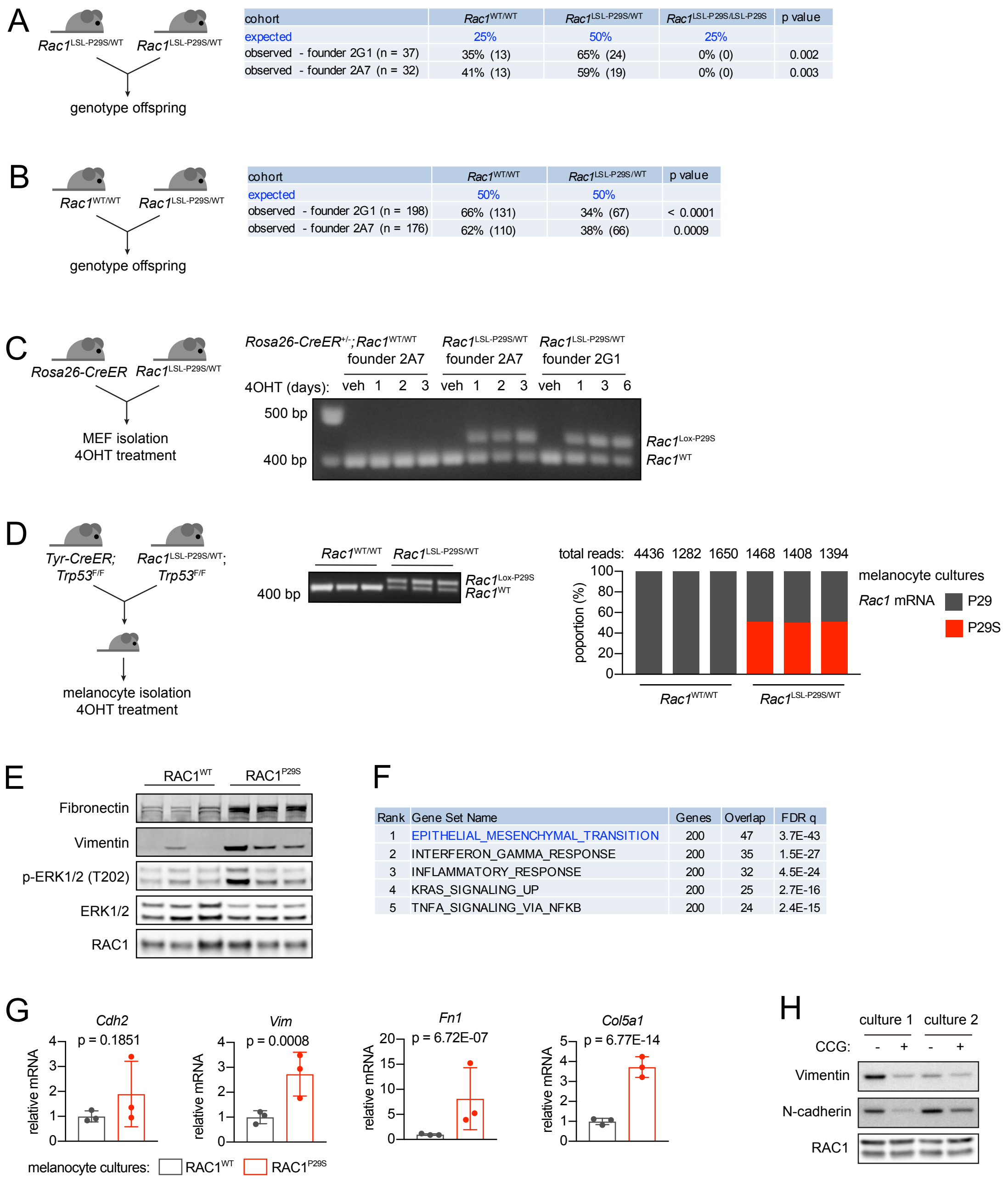

Figure S3. Effect of endogenous RAC1^{P29S} on activation of AKT and SRF/MRTF in melanocytes and induction of mesenchymal phenotype.

Related to Figure 3.

(A) Breeding scheme and genotype table indicating developmental effect of the homozygous Rac1$^{LSL\text{-}P29S}$ allele. Absolute numbers of genotyped offspring are shown between parentheses. Chi-square test was used to statistically assess differences between expected and observed genotypes.

(B) Breeding scheme and genotype table indicating developmental effect of the heterozygous Rac1$^{LSL\text{-}P29S}$ allele. Absolute numbers of genotyped offspring are shown

between parentheses. Chi-square test was used to statistically assess differences between expected and observed genotypes.
(C) Breeding scheme and recombination of the *Rac1*$^{LSL-P29S}$ allele in mouse embryonic fibroblasts (MEFs). MEFs were isolated and recombined using daily refreshed 4OHT at 1 μM. Genomic DNA was isolated and used in the recombination PCR assay.
(D) Breeding scheme and recombination of Rac1$^{LSL-P29S}$ allele in melanocytes from neonatal mice. Melanocytes were isolated and recombined between the first and second passage using 4OHT (1 μM). Genomic DNA was subjected to a recombination PCR assay and mRNA was sequenced to obtain read counts of *Rac1*^{P29} versus *Rac1*^{P29S}.
(E) Phosphorylation of ERK1/2 and protein expression of EMT markers in melanocytes with endogenous RAC1^{P29S}. Immunoblot of 3 independent cultures per genotype is shown.
(F) Pathway enrichment analysis of gene expression differences in melanocytes with endogenous RAC1^{P29S} versus RAC1^{WT}. RNA from independent melanocyte cultures was sequenced (n = 3 per genotype). Overlap was calculated between genes upregulated in RAC1^{P29S} melanocytes (log2 fold-change > 4 and adjusted p value < 0.05, n = 799 genes) and the curated "hallmark" gene set collection from the MSigDB database (Broad).
(G) Normalized mRNA read counts of EMT markers in melanocytes with endogenous RAC1^{P29S}. Bars represent means ± SD (n = 3 independent melanocyte cultures per group). For statistical comparison of groups, Wald test was applied in combination with Benjamini and Hochberg correction.
(H) Effect of inhibition of the SRF/MRTF pathway on expression of the mesenchymal markers vimentin and N-cadherin in melanocytes with endogenous RAC1^{P29S}. Cells were treated with 25 μM CCG-203971 for 24 hr, lysed and processed for immunoblotting with the indicated antibodies.

A

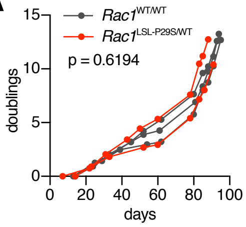

B

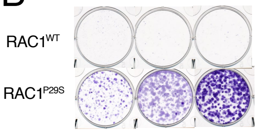

C

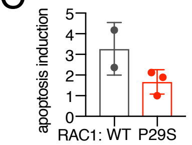

D

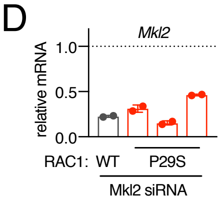

E

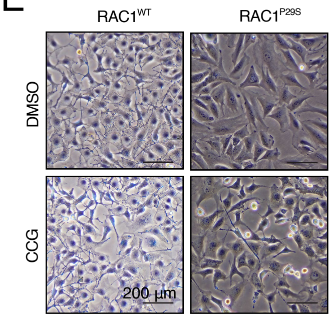

F

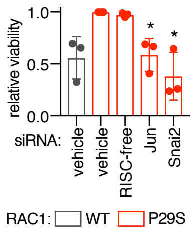

G

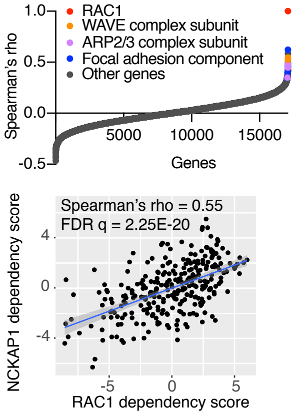

rank	gene	rho	FDR q	annotation
1	RAC1	1	0	RAC1
2	TLN1	0.62	2.02E-28	focal adhesions
3	RAPGEF1	0.58	2.56E-43	
4	NCKAP1	0.55	2.25E-20	WAVE subunit
5	CYFIP1	0.52	1.63E-18	WAVE subunit
6	CRKL	0.50	9.06E-29	
7	BCAR1	0.49	1.11E-27	
8	ARPC2	0.48	3.91E-27	ARP2/3 subunit
9	PTK2	0.47	3.34E-25	focal adhesions
10	SRP54	0.46	1.22E-13	
11	RPN2	0.45	2.72E-13	
12	ACTR3	0.45	6.06E-23	ARP2/3 subunit
13	ACTR2	0.45	6.06E-23	ARP2/3 subunit
14	FERMT2	0.45	6.86E-13	focal adhesions
15	PI4KAP1	0.43	1.62E-20	
16	PI4KA	0.42	3.75E-20	
17	OSTC	0.41	4.16E-11	
18	CRK	0.41	6.86E-19	
19	INHBE	0.41	1.73E-18	
20	CARD8	0.41	1.34E-10	

J

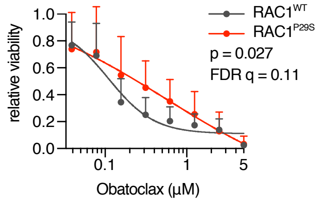

K

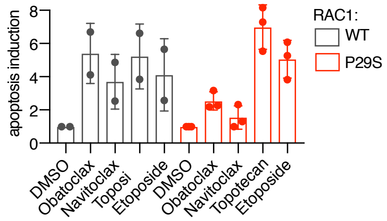

H

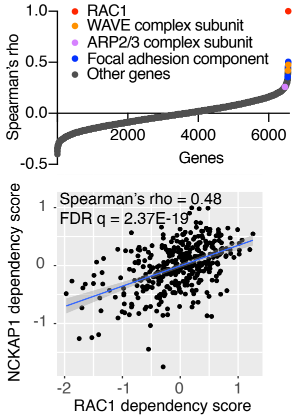

rank	gene	rho	FDR q	annotation
1	RAC1	1	0	RAC1
2	TLN1	0.51	3.21E-22	focal adhesions
3	RAPGEF1	0.50	3.21E-22	
4	NCKAP1	0.48	2.37E-19	WAVE subunit
5	ITGAV	0.46	7.78E-18	focal adhesions
6	CRKL	0.45	3.46E-17	
7	BCAR1	0.44	1.50E-16	
8	TEAD1	0.43	1.13E-15	
9	ABI1	0.42	1.56E-14	WAVE subunit
10	POU4F2	0.41	4.46E-14	
11	PKN2	0.40	1.35E-13	
12	YAP1	0.39	1.13E-12	
13	DLG5	0.39	1.22E-12	
14	CEL	0.37	1.02E-10	
15	ITGB5	0.36	2.04E-10	focal adhesions
16	CNGA4	0.36	3.91E-10	
17	CYR61	0.36	4.73E-10	focal adhesions
18	SLC39A10	0.35	1.41E-09	
19	PATZ1	0.33	7.17E-09	
20	DAD1	0.33	8.27E-09	

L

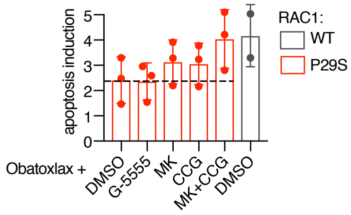

I

cell line	*RAC1*	*BRAF*	*NRAS*	*NF1*
SK-MEL-2	WT	WT	Q61R	WT
SK-MEL-5	WT	V600E	WT	WT
LOX-IMVI	WT	V600E, I208V	WT	Q1174*
A2058	WT	V600E	WT	WT
Colo-792	WT	WT	WT	W1236R
C32	WT	V600E	WT	WT
A375	WT	V600E	WT	WT
WM3060	P29S	WT	Q61K	WT
WM1791C	P29S	WT	WT	WT
IGR1	P29S	V600E, V600M	WT	WT
YUHEF	P29S	WT	WT	Q853*
YUSOC	P29S	WT	WT	W336*, E337K
YURIF	P29S	V600K	WT	WT
YUTOGS	P29S	WT	WT	L446F, K2535*

Figure S4. Discovery of AKT and MRTF dependencies in cells with endogenous RAC1^{P29S}.

Related to Figure 4.

(A) Population doublings of independent melanocyte cultures from *Rac1*$^{WT/WT}$ and *Rac1*$^{LSL\text{-}P29S/WT}$ mice crossed with *Tyr-CreER*$^{+/-}$;*Trp53*$^{F/F}$ mice. Cultures were treated with 4OHT at 1 μM from the first to second passage (9-15 days) and counted at every passage with the Countess Automated Cell Counter (Thermo Fisher Scientific). Two-way ANOVA was used to compare mean growth curves, with the p value for the genotype effect indicated.

(B) Clonogenicity of melanocytes with endogenous RAC1^{P29S}. Fifteen-hundred cells per well were seeded onto 6-well plates and cultured in growth medium, refreshed after 3 days. Seven days post-seeding, cells were fixed and stained with crystal violet. Representative wells from 3 independent melanocyte cultures per genotype are shown.

(C) Induction of apoptosis in melanocytes with endogenous RAC1^{P29S} exposed to ultra-low adhesion and low serum. Cells were cultured in 0.25% FCS without TPA and plated onto ultra-low adhesion plates. Apoptosis was normalized to total cell viability and values at 48 hr were compared to values shortly after seeding. Bars represent means ± SD (n = 2-3 cultures per genotype, each assayed in 3 independent experiments).

(D) Genetic knock-down efficiency in mouse melanocytes. Three cultures with endogenous RAC1^{P29S} and 1 RAC1^{WT} culture were transfected with an Mkl2 siRNA pool. Two days post-transfection, mRNA expression was determined using qPCR. Bars represent means ± SD (n = 2 experimental replicates).

(E) Morphological effects of chemical SRF/MRTF pathway inhibition in mouse melanocytes with endogenous RAC1^{P29S}. Cells were cultured in growth medium and treated with vehicle (DMSO) or CCG-203971 at 10 μM for 48 hr. Representative light microscopy photographs are shown.

(F) Effect of depletion of transcription factors involved in EMT on survival ability of melanocytes with endogenous RAC1^{P29S}. Cells were transfected with the indicated siRNAs and cultured in growth factor-reduced medium, which contained 0.25% FCS without TPA. Seventy-two hr post-transfection, viability was quantified using CellTiter-Blue. Bars represent means ± SD (n = 3 independent cell cultures, each value is a mean from 3 independent experiments); t-test versus RISC-free was used with Holm-Sidak correction for multiple testing; *, $p < 0.05$.

(G) Co-dependencies in human cancer cell lines dependent on RAC1, studied using publicly available shRNA screening data of human cancer cell lines (Tsherniak et al., 2017). Cell lines were part of the Cancer Cell Line Encyclopedia (CCLE). Top left figure: for each gene in the library (n = 17098 genes, average ~6 shRNAs/gene), correlation with RAC1 was tested with Spearman's rho, using dependency scores of different cell lines (n = 501) as data points. Bottom left figure: correlation of RAC1 and NCKAP1 dependency scores is shown as an example, where each dot represents a cell line. The blue line is a linear regression fit with the grey band representing the 95% confidence interval. Table: the top 20 RAC1 co-dependencies.

(H) The analysis presented in panel F was repeated using an independent screening dataset of human cancer cell lines (McDonald et al., 2017). These screens were

performed on 383 cell lines also part of the CCLE, using a shRNA library targeting 7837 genes (average 20 shRNAs/gene).

(I) Table of human melanoma cell line panel with endogenous $RAC1^{P29S}$ and $RAC1^{WT}$. Genotypes for *RAC1*, *BRAF*, *NRAS* and *NF1* are shown.

(J) Comparison of human melanoma cell lines with $RAC1^{WT}$ or $RAC1^{P29S}$ response to inhibition of the pro-survival Bcl-2 family with obatoclax. Viability was determined using CellTiter-Glo and normalized to DMSO vehicle. Values represent means ± SD (n = 7 cell lines per genotype, for each cell line the mean from 2 independent experiments was used). Two-way ANOVA was used to compare curves, with the p value for the genotype effect indicated. Benjamini-Hochberg correction for multiple comparisons was performed to produce FDR *q* values.

(K) Effect of $RAC1^{P29S}$ on induction of apoptosis by the anti-apoptotic Bcl-2 family inhibitors obatoclax and navitoclax, and the DNA damaging agents topotecan and etoposide. Mouse melanocytes with endogenous $RAC1^{P29S}$ or $RAC1^{WT}$ were cultured in growth medium and treated with DMSO vehicle, obatoclax at 500 nM, navitoclax at 5 μM, topotecan at 625 nM or etoposide at 1250 nM. Three days after treatment, apoptosis was measured and normalized to total cell viability. Values represent means ± SD (n = 2-3 melanocyte cultures per genotype, for each cell line the mean from 2-3 independent experiments was used).

(L) Effect on $RAC1^{P29S}$-induced protection against apoptosis of inhibition of the SRF/MRTF and AKT pathways. Mouse melanocytes with endogenous $RAC1^{P29S}$ or $RAC1^{WT}$ were cultured in growth medium and treated with obatoclax at 500 nM in combination with DMSO vehicle, the PAK inhibitor G-5555 at 2 μM, the AKT inhibitor MK-2206 at 2 μM or the SRF/MRTF pathway inhibitor CCG-203971 at 10 μM. Three days after treatment, apoptosis was measured and normalized to total cell viability. Because of its transcriptional mechanism of action, cells were pre-treated with CCG-203971 for 3 days before inducing apoptosis with obatoclax. Values represent means ± SD (n = 2-3 melanocyte cultures per genotype).

A

cohort	*Rac1*$^{WT/WT}$	*Rac1*$^{LSL-P29S/WT}$	p value
expected	50%	50%	
observed - founder 2G1 (n = 26)	100% (26)	0% (0)	< 0.0001
observed - founder 2A7 (n = 30)	100% (30)	0% (0)	< 0.0001

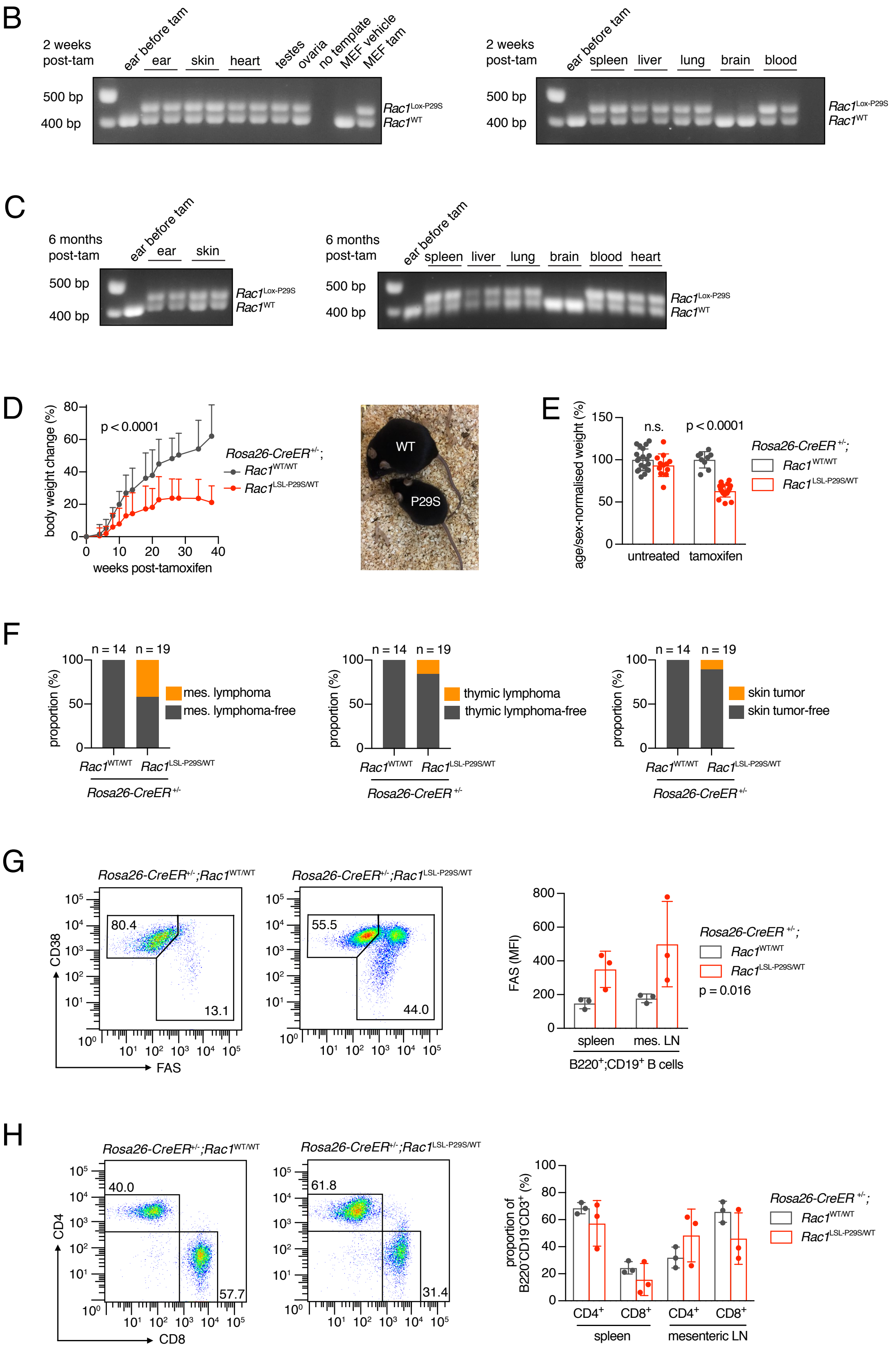

Figure S5. Effect of ubiquitous expression of $RAC1^{P29S}$ *in vivo* on B cell lymphoma formation.

Related to Figure 5.

(A) Table demonstrating effects on embryonic when $RAC1^{P29S}$ is expressed in the mouse germline by breeding *PGK-Cre* females with males carrying the $Rac1^{LSL\text{-}P29S}$. Live pups were genotyped at weaning age for this analysis. We did not observe abnormalities in the survival of neonatal mice. The p values from Chi-square tests are indicated.

(B) Recombination PCR assay using genomic DNA from various tissues of two *Rosa26-CreER*$^{+/-}$;*Rac1*$^{LSL\text{-}P29S/WT}$ mice that were sacrificed 2 weeks after starting treatment of tamoxifen per oral gavage. For control purposes we included genomic DNA from mouse embryonic fibroblasts (MEFs) recombined in vitro.

(C) Recombination PCR assay using genomic DNA from various tissues of two *Rosa26-CreER*$^{+/-}$;*Rac1*$^{LSL\text{-}P29S/WT}$ mice that were sacrificed 6 months after starting treatment of tamoxifen per oral gavage.

(D) Effect on weight gain in mice with ubiquitous $RAC1^{P29S}$. Weight curves following tamoxifen treatment are shown next to a representative photo of littermates with *Rosa26CreER*$^{+/-}$;*Rac1*$^{WT/WT}$ (WT) or *Rosa26CreER*$^{+/-}$;*Rac1*$^{LSL\text{-}P29S/WT}$ (P29S) genotype. Values represent means ± SD (n = 7-17 mice). Two-way ANOVA was used to compare curves, with the p value for the genotype effect indicated.

(E) Effect on body weight of *Rosa26CreER*$^{+/-}$;*Rac1*$^{LSL\text{-}P29S/WT}$ mice after treatment with tamoxifen. Values represent means ± SD (n = 8-20 mice). To permit comparison of mice with different ages and sex, animals were pooled in sex-specific 6-week age groups for normalization. Mann-Whitney testing was used to compare genotypes; n.s., not statistically significant.

(F) Frequency of different tumor types found in ageing mice with ubiquitous $RAC1^{P29S}$.

(G) Effect on transformation of the B cell compartment ($B220^+$;$CD19^+$) in mesenteric lymphoma of mice with ubiquitous $RAC1^{P29S}$. Left panel: representative flow cytometric plots of B cells from a mesenteric lymph node of a *Rosa26-CreER*$^{+/-}$;*Rac1*$^{WT/WT}$ mouse compared to B cells from a mesenteric lymphoma of a *Rosa26-CreER*$^{+/-}$;*Rac1*$^{LSL\text{-}P29S/WT}$ mouse, demonstrating emergence of a FAS-high population. Right panel: mean FAS expression in B cells from the spleen and mesenteric lymph node (mLN) or mesenteric lymphoma. Bars represent means ± SD (n = 3 animals). Two-way ANOVA was used to compare means, with the p value for the genotype effect indicated. MFI, mean fluorescence intensity.

(H) Effect on the T cell compartment ($B220^-$;$CD19^-$;$CD3^+$)in mesenteric lymphomas of mice with ubiquitous $RAC1^{P29S}$. Left panel: representative flow cytometric plots of T cells from a mesenteric lymph node of a *Rosa26-CreER*$^{+/-}$;*Rac1*$^{WT/WT}$ mouse compared to B cells from a mesenteric lymphoma of a *Rosa26-CreER+/-;Rac1*$^{LSL\text{-}P29S/WT}$ mouse. Right panel: proportion of $CD4^+$ and $CD8^+$ populations in T cells from the spleen and mesenteric lymph node or mesenteric lymphoma. Bars represent means ± SD (n = 3 animals).

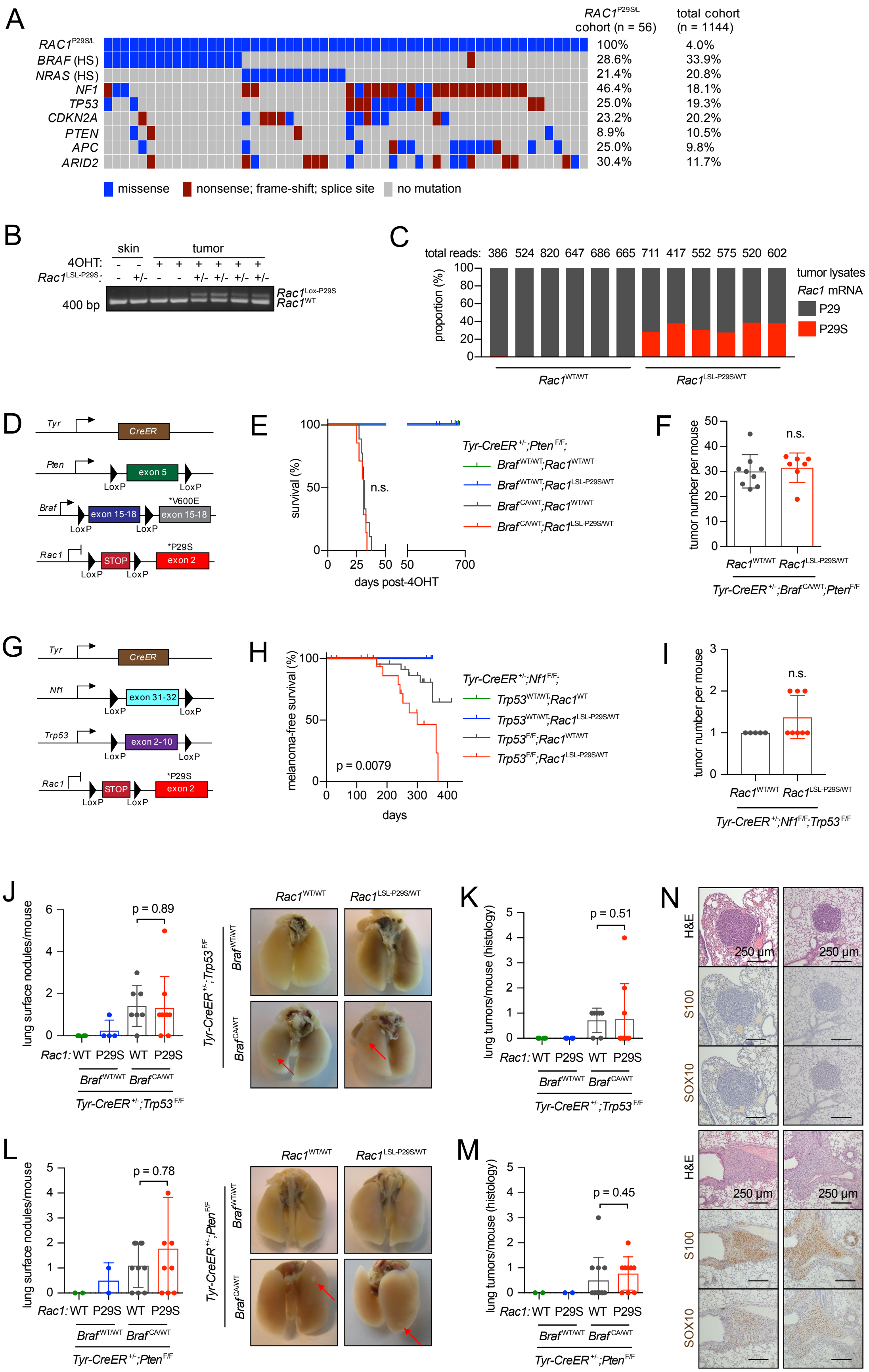
A
RAC1 P29S/L cohort (n = 56)
total cohort (n = 1144)
RAC1 P29S/L 100% 4.0%
BRAF (HS) 28.6% 33.9%
NRAS (HS) 21.4% 20.8%
NF1 46.4% 18.1%
TP53 25.0% 19.3%
CDKN2A 23.2% 20.2%
PTEN 8.9% 10.5%
APC 25.0% 9.8%
ARID2 30.4% 11.7%
missense
nonsense; frame-shift; splice site
no mutation
B
skin
tumor
4OHT:
Rac1 LSL-P29S:
400 bp
Rac1 Lox-P29S
Rac1 WT
C
total reads: 386 524 820 647 686 665 711 417 552 575 520 602
proportion (%)
tumor lysates
Rac1 mRNA
P29
P29S
Rac1 WT/WT
Rac1 LSL-P29S/WT
D
Tyr
CreER
Pten
exon 5
LoxP
Braf
exon 15-18
*V600E
Rac1
STOP
exon 2
*P29S
E
survival (%)
n.s.
days post-4OHT
Tyr-CreER +/-;Pten F/F;
Braf WT/WT;Rac1 WT/WT
Braf WT/WT;Rac1 LSL-P29S/WT
Braf CA/WT;Rac1 WT/WT
Braf CA/WT;Rac1 LSL-P29S/WT
F
tumor number per mouse
n.s.
Rac1 WT/WT
Rac1 LSL-P29S/WT
Tyr-CreER +/-;Braf CA/WT;Pten F/F
G
Tyr
CreER
Nf1
exon 31-32
Trp53
exon 2-10
Rac1
STOP
exon 2
*P29S
LoxP
H
melanoma-free survival (%)
p = 0.0079
days
Tyr-CreER +/-;Nf1 F/F;
Trp53 WT/WT;Rac1 WT
Trp53 WT/WT;Rac1 LSL-P29S/WT
Trp53 F/F;Rac1 WT/WT
Trp53 F/F;Rac1 LSL-P29S/WT
I
tumor number per mouse
n.s.
Rac1 WT/WT
Rac1 LSL-P29S/WT
Tyr-CreER +/-;Nf1 F/F;Trp53 F/F
J
lung surface nodules/mouse
p = 0.89
Rac1: WT P29S WT P29S
Braf WT/WT
Braf CA/WT
Tyr-CreER +/-;Trp53 F/F
Rac1 WT/WT
Rac1 LSL-P29S/WT
K
lung tumors/mouse (histology)
p = 0.51
Rac1: WT P29S WT P29S
Braf WT/WT
Braf CA/WT
Tyr-CreER +/-;Trp53 F/F
L
lung surface nodules/mouse
p = 0.78
Rac1: WT P29S WT P29S
Braf WT/WT
Braf CA/WT
Tyr-CreER +/-;Pten F/F
Rac1 WT/WT
Rac1 LSL-P29S/WT
M
lung tumors/mouse (histology)
p = 0.45
Rac1: WT P29S WT P29S
Braf WT/WT
Braf CA/WT
Tyr-CreER +/-;Pten F/F
N
H&E
S100
SOX10
250 µm

Figure S6. Effect of melanocytic expression of RAC1^{P29S} *in vivo* on tumorigenesis. Related to Figure 6.

(A) Mutational landscape of RAC1-mutant human cutaneous melanoma. Sequencing data from multiple cohorts were compiled. Only cases with *RAC1*^{P29S} (n = 51) or *RAC1*^{P29L} (n = 5) are shown here. For *BRAF* and *NRAS*, only the following hotspot (HS) mutations are indicated blue: *BRAF* V600E/K and K601E; *NRAS* Q61K/L/R/H. For the remaining genes, any missense mutation is indicated blue.

(B) Recombination of the *Rac1*$^{LSL\text{-}P29S}$ allele in normal skin and tumor lysates. Genomic DNA was isolated from dorsal skin not bearing any tumors and BRAF^{V600E} or BRAF^{V600E};PTEN$^{+/-}$ tumors and used in a recombination PCR assay.

(C) Messenger RNA read counts of *Rac1*^{P29} versus *Rac1*^{P29S} in *Tyr-CreER*$^{+/-}$*;Pten*$^{F/WT}$*;Braf*$^{CA/WT}$ tumor lysates, obtained by next-generation sequencing.

(D) Schematic of the *Tyr-CreER*, *Pten*^{F}, *Braf*^{CA} and *Rac1*$^{LSL\text{-}P29S}$ allele combination.

(E) Effect of RAC1^{P29S} expression on melanomagenesis in a BRAF^{V600E};PTEN-null melanoma model. Survival curves (tumor-associated death) of *Tyr-CreER*$^{+/-}$*;Pten*$^{F/F}$*;Braf*$^{CA/WT}$*;Rac1*$^{WT/WT}$ mice and *Tyr-CreER*$^{+/-}$*;Pten*$^{F/F}$*;Braf*$^{CA/WT}$*;Rac1*$^{LSL\text{-}P29S/WT}$ mice were compared using log-rank testing (Mantel-Cox).

(F) Effects of RAC1^{P29S} on tumor number in *Tyr-CreER*$^{+/-}$*;Pten*$^{F/F}$*;Braf*$^{CA/WT}$ mice. Bars represent means ± SD (n = 7-9 mice per group). Groups were compared using an unpaired two-tailed t-test.

(G) Schematic of the *Tyr-CreER*, *Nf1*^{F}, *Trp53*^{F} and *Rac1*$^{LSL\text{-}P29S}$ allele combination.

(H) Effect of RAC1^{P29S} expression on melanomagenesis in an NF1-null;p53-null melanoma model. Melanoma-free survival curves of *Tyr-CreER*$^{+/-}$*;Nf1*$^{F/F}$*;Trp53*$^{F/F}$*;Rac1*$^{WT/WT}$ mice and *Tyr-CreER*$^{+/-}$*;Nf1*$^{F/F}$*;Trp53*$^{F/F}$*;Rac1*$^{LSL\text{-}P29S/WT}$ mice were compared using log-rank testing (Mantel-Cox), with the p value indicated.

(I) Effects of RAC1^{P29S} on tumor number in *Tyr-CreER*$^{+/-}$*;Nf1*$^{F/F}$*;Trp53*$^{F/F}$ mice. Bars represent means ± SD (n = 5-8 mice per group). Groups were compared using the Mann-Whitney test.

(J) Effects of RAC1^{P29S} on lung metastasis in the BRAF^{V600E};p53-null melanoma model. Lung surface nodules were counted using a dissection microscope. Photos of representative lungs are shown. An unpaired two-tailed t-test was used for statistical comparison.

(K) Effects of RAC1^{P29S} on lung metastasis in a BRAF^{V600E};p53-null melanoma model, determined by histological analysis of lungs for (micro)metastasis. The whole lungs were fixed, sectioned and three levels were analyzed by a blinded pathologist. Mann-Whitney test was used for statistical comparison.

(L) Effects of RAC1^{P29S} on lung metastasis in a BRAF^{V600E};PTEN-null melanoma model, determined by counting lung surface nodules using a dissection microscope. Photos of representative lungs are shown. Mann-Whitney test was used for statistical comparison.

(M) Effects of RAC1^{P29S} on lung metastasis in a BRAF^{V600E};PTEN-null melanoma model, determined by histological analysis of lungs for (micro)metastases. The whole lungs were fixed, sectioned and three levels were analyzed by a blinded pathologist. An unpaired two-tailed t-test was used for statistical comparison.

(N) Representative H&E, S100 and SOX10-stained sections of two primary broncho-alveolar tumors (upper six micrographs) and two genuine melanoma metastases (lower six micrographs).

Wherever used, error bars represent means ± SD.

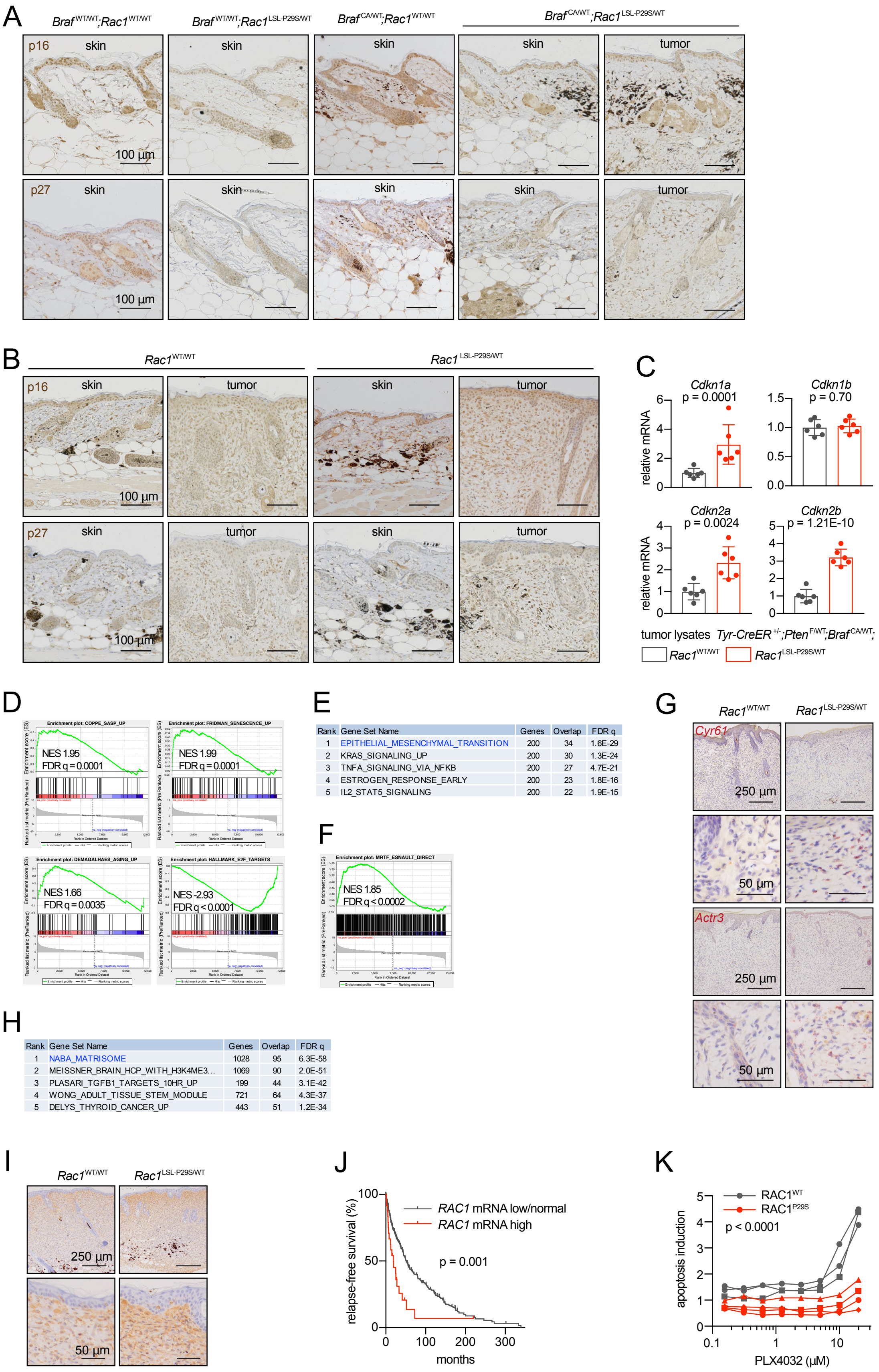

Rank	Gene Set Name	Genes	Overlap	FDR q
1	EPITHELIAL_MESENCHYMAL_TRANSITION	200	34	1.6E-29
2	KRAS_SIGNALING_UP	200	30	1.3E-24
3	TNFA_SIGNALING_VIA_NFKB	200	27	4.7E-21
4	ESTROGEN_RESPONSE_EARLY	200	23	1.8E-16
5	IL2_STAT5_SIGNALING	200	22	1.9E-15

Rank	Gene Set Name	Genes	Overlap	FDR q
1	NABA_MATRISOME	1028	95	6.3E-58
2	MEISSNER_BRAIN_HCP_WITH_H3K4ME3...	1069	90	2.0E-51
3	PLASARI_TGFB1_TARGETS_10HR_UP	199	44	3.1E-42
4	WONG_ADULT_TISSUE_STEM_MODULE	721	64	4.3E-37
5	DELYS_THYROID_CANCER_UP	443	51	1.2E-34

Figure S7. RAC1^{P29S} effects on drug resistance and mesenchymal phenotype in BRAF-driven melanoma.

Related to Figure 7.

(A) Expression of senescence markers in the skin and tumors of mice from the BRAF^{V600E} melanoma model. Representative sections stained with p16 and p27 (IHC) are shown. In addition to the genotype indicated above the panels, all mice were *Tyr-CreER*$^{+/-}$.

(B) Expression of senescence markers in the skin and tumors of mice from the BRAF^{V600E};PTEN-hemizygous melanoma model. Representative sections stained with p16 and p27 (IHC) are shown. In addition to the genotype indicated above the panels, all mice were *Tyr-CreER*$^{+/-}$; *Pten*$^{F/WT}$; *Braf*$^{CA/WT}$.

(C) Normalized mRNA read counts of senescence markers in tumor lysates from *Tyr-CreER*$^{+/-}$*;Pten*$^{F/WT}$*;Braf*$^{CA/WT}$ mice. Bars represent means ± SD (n = 6 tumors from 5-6 animals per group). For statistical comparison of groups, Wald test was applied in combination with the Benjamini and Hochberg correction for multiple comparisons.

(D) GSEA using gene signatures of the senescence-associated secretory phenotype (SASP), senescence, ageing and E2F targets, applied on RNA-seq data of tumor lysates from *Tyr-CreER*$^{+/-}$*;Pten*$^{F/WT}$*;Braf*$^{CA/WT}$*;Rac1*$^{LSL\text{-}P29S/WT}$ mice versus tumor lysates from *Tyr-CreER*$^{+/-}$*;Pten*$^{F/WT}$*;Braf*$^{CA/WT}$*;Rac1*$^{WT/WT}$ mice (n = 6 tumors from 5-6 animals per group).

(E) Pathway enrichment analysis of gene expression differences induced by RAC1^{P29S} in tumors. RNA-seq data from *Tyr-CreER*$^{+/-}$*;Pten*$^{F/WT}$*;Braf*$^{CA/WT}$ mice was used (n = 6 tumors from 5-6 animals per group). Overlap was calculated between genes upregulated in RAC1^{P29S} tumors compared to RAC1^{WT} tumors (log2 fold-change > 1 and adjusted p value < 0.05, n = 536 genes) and the curated “hallmark” gene set collection from the MSigDB database (Broad).

(F) Effect on the SRF/MRTF transcriptional program in RAC1^{P29S} tumors. GSEA was performed using SRF/MRTF/TCF gene sets (Esnault et al., 2014) in combination with RNA-seq of tumor lysates from *Tyr-CreER*$^{+/-}$*;Pten*$^{F/WT}$*;Braf*$^{CA/WT}$*;Rac1*$^{LSL\text{-}P29S/WT}$ mice versus tumor lysates from *Tyr-CreER*$^{+/-}$*;Pten*$^{F/WT}$*;Braf*$^{CA/WT}$*;Rac1*$^{WT/WT}$ mice (n = 6 tumors from 5-6 animals per group). GSEA plot for direct targets of MRTF is shown.

(G) mRNA in situ hybridization of canonical SRF/MRTF targets *Cyr61* (top four) and *Actr3* (bottom four). Tumors from *Tyr-CreER*$^{+/-}$*;Pten*$^{F/WT}$*;Braf*$^{CA/WT}$*;Rac1*$^{LSL\text{-}P29S/WT}$ mice versus *Tyr-CreER*$^{+/-}$*;Pten*$^{F/WT}$*;Braf*$^{CA/WT}$*;Rac1*$^{WT/WT}$ mice were used (n = 4 animals from each genotype) with one representative micrograph from each genotype shown.

(H) Pathway enrichment analysis of gene expression differences induced by RAC1^{P29S} in tumors. RNA-seq data from *Tyr-CreER*$^{+/-}$*;Pten*$^{F/WT}$*;Braf*$^{CA/WT}$ mice was used (n = 6 tumors from 5-6 animals per group). Overlap was calculated between genes upregulated in RAC1^{P29S} tumors compared to RAC1^{WT} tumors (log2 fold-change > 1 and adjusted p value < 0.05, n = 536 genes) and the curated C2 gene set collection (n = 4838 gene sets) from the MSigDB database (Broad).

(I) Immunostaining for vimentin expression in tumors with RAC1^{P29S}. Tumors from *Tyr-CreER*$^{+/-}$*;Pten*$^{F/WT}$*;Braf*$^{CA/WT}$ mice (n = at least 4 tumors per *Rac1* genotype) were used for immunohistochemistry, with representative micrographs shown.

(J) *RAC1* mRNA overexpression effects on relapse-free survival in patients with melanoma. Publicly available data from the TCGA cutaneous melanoma cohort was

used (n = 316 patients). Twenty-four patients met the *RAC1* mRNA high threshold, set at > 4 SDs above the mean. Groups were statistically compared using log-rank testing (Mantel-Cox), with the p value indicated.

(K) Comparison of mouse melanoma cell lines with $RAC1^{P29S}$ or $RAC1^{WT}$ on apoptosis induction by BRAF inhibition. Four $RAC1^{P29S}$ and 3 $RAC1^{WT}$ tumor cell cultures from *Tyr-CreER*$^{+/-}$*;Tp53*$^{F/F}$*;Braf*$^{CA/WT}$ mice were treated with PLX4032 (vemurafenib) and assayed for apoptosis 24 hr later. Apoptosis induction relative to DMSO control is shown. Values represent means from 2 independent experiments. Two-way ANOVA was used to compare mean growth curves, with the p value for the genotype effect indicated.
